# Transcranial Ultrasound Stimulation Pulsed at 40 Hz Improves Cognition and Neuroinflammation in Female Mice with Alzheimer’s Disease

**DOI:** 10.34133/research.1244

**Published:** 2026-04-20

**Authors:** Shasha Yi, Junjie Zou, Xiaofei He, Houminji Chen, Zhengrong Lin, Liying Zhang, Yiyue Zhu, Zejie Zuo, Zehao Chen, Xiquan Hu, Lili Niu

**Affiliations:** ^1^ Institute of Biomedical and Health Engineering, Shenzhen Institutes of Advanced Technology, Chinese Academy of Sciences, Shenzhen 518055, China.; ^2^ Department of Rehabilitation Medicine, The Third Affiliated Hospital, Sun Yat-sen University, Guangzhou 510630, China.

## Abstract

Recent advances in transcranial ultrasound stimulation (TUS) pulsed at 40 Hz have demonstrated the potential to ameliorate cognitive deficits in mouse models of Alzheimer’s disease. However, technical barriers remain as general anesthesia is required for mice, which restricts the accurate elucidation of biological mechanisms and behavioral effects under awake physiological conditions. Here, we report a wearable, free-moving ultrasound stimulation system that delivers TUS pulsed at 40 Hz to female 5xFAD transgenic mice to systematically evaluate the behavioral outcomes and underlying mechanistic pathways. Among the treatment groups, a 14-d regimen at an acoustic intensity of 2.14 W/cm^2^ yielded the optimal cognitive outcome in Alzheimer’s disease mice, which was consistently verified across Y-maze and Morris water maze tests. Additionally, this group showed reduced Aβ plaque deposition and increased plaque-associated microglial activity. Furthermore, enhanced gamma oscillations in the hippocampus were detected following treatment. RNA sequencing revealed modulation of innate immune and inflammatory pathways. Corresponding molecular analysis demonstrated a marked down-regulation in *RIPK1*, phosphorylated *NF-κB*, and necroptosis markers, alongside reductions in key pro-inflammatory cytokines (*IL-6*, *IL-1β*, and *TNF-α*). Collectively, our findings suggest that the cognitive improvement observed after treatment with TUS pulsed at 40 Hz may be linked to the modulation of neuroinflammatory and necroptotic pathways, possibly involving *RIPK1*/*NF-κB* signaling.

## Introduction

Alzheimer’s disease (AD), a progressive neurodegenerative condition, represents the most common cause of dementia [1,2]. Currently, over 50 million individuals worldwide are affected by AD [3]. Current projections point to a rise in the global prevalence of AD to over 150 million cases by 2050 [4,5], posing important health challenges. AD is manifested through the aberrant extracellular deposition of amyloid-β (Aβ plaques) alongside the intracellular accumulation of hyperphosphorylated tau protein forming neurofibrillary tangles [6]. Furthermore, AD exhibits synaptic and neuronal loss, reactive glial cell proliferation, and neuroinflammation [7]. Numerous therapeutic efforts have therefore targeted these key pathogenic drivers, advancing multiple candidates into clinical trials. Despite this concerted effort, a substantial proportion of these candidates have failed to meaningfully slow AD progression [8–10].

Neuronal activity is characterized by rhythmic oscillations across distinct frequency bands. Among these, gamma-band neural oscillations are crucial for cognitive processes [11,12]. A well-documented observation in AD is the pervasive reduction in gamma-band oscillatory activity in both clinical and preclinical settings [13–15]. This compelling evidence has prompted the exploration of targeted gamma oscillation induction as a novel intervention for AD. Pioneering work by Tsai and colleagues [16] provided initial evidence that 40-Hz flickering light reduces amyloid plaque burden in the visual cortex of AD mice. Further research by the same team revealed that multisensory 40-Hz stimulation, combining both visual and auditory modalities, not only reduced plaque deposition in the auditory and visual cortices, hippocampus, and medial prefrontal cortex but also significantly rescued memory deficits in 5xFAD mice [17]. Beyond sensory stimulation, other physical techniques for inducing 40-Hz brain oscillations, including electrical stimulation [18], magnetic stimulation [19], and vibrotactile stimulation [20], have also demonstrated promising therapeutic efficacy in ameliorating AD pathological hallmarks and cognitive deficits. Taken together, these findings support the augmentation of gamma oscillatory activity as a viable strategy to counteract both pathological progression and cognitive decline in AD.

However, existing interventional strategies struggle to achieve direct stimulation of deep-brain-region-related memory under noninvasive conditions. Consequently, this limitation has spurred the need for a technology that can precisely target these areas to induce gamma oscillations. Transcranial ultrasound stimulation (TUS), a neuromodulation tool with unique spatial targeting capabilities, has demonstrated neuroprotective effects against cognitive impairment in both animal and human studies [21]. Notably, TUS administered at a 40-Hz pulse repetition frequency (PRF) has garnered attention for its ability to specifically entrain gamma-band neural oscillations, offering potential therapeutic benefits. Previous studies have established that TUS pulsed at 40 Hz induces gamma activity, activates microglia, promotes their migration to Aβ plaques, and clears amyloid burden in AD mice [22] while also restoring normal cross-frequency coupling [23]. However, translating this approach into clinical practice requires addressing several methodological and mechanistic challenges. Beyond the 40 Hz of PRF, systematic optimization of other stimulation parameters—such as acoustic intensity, duty cycle, and treatment duration—is critical for maximizing efficacy and safety. Furthermore, the pervasive distribution of Aβ pathology necessitates stimulation strategies that can target broader brain regions, highlighting the need for wearable devices capable of large-scale neuromodulation in freely moving subjects. Therefore, the integration of parameter optimization with wearable platforms will facilitate the exploration of the potential mechanisms underlying the therapeutic effects of TUS pulsed at 40 Hz.

In this study, we developed a head-mounted planar ultrasound transducer that enables large-scale neuromodulation in freely behaving mice. A 14-d regimen of TUS pulsed at 40 Hz (*I*_spta_ = 2.14 W/cm^2^) significantly alleviated cognitive deficits and reduced Aβ deposition in 5xFAD mice. These benefits were concurrent with a multi-level restorative response, including the restoration of hippocampal gamma oscillations and synaptic integrity, alongside attenuated neuroinflammation involving the *RIPK1*/*NF-κB* pathway and modulated microglial function. These coordinated changes ultimately supported cognitive improvement. Together, our findings establish TUS pulsed at 40 Hz as a novel, noninvasive strategy that targets core AD pathologies, highlighting its promising translational potential for cognitive disorders.

## Results

### TUS treatment rescues cognitive deficits in 5xFAD mice

**Fig. 1.**
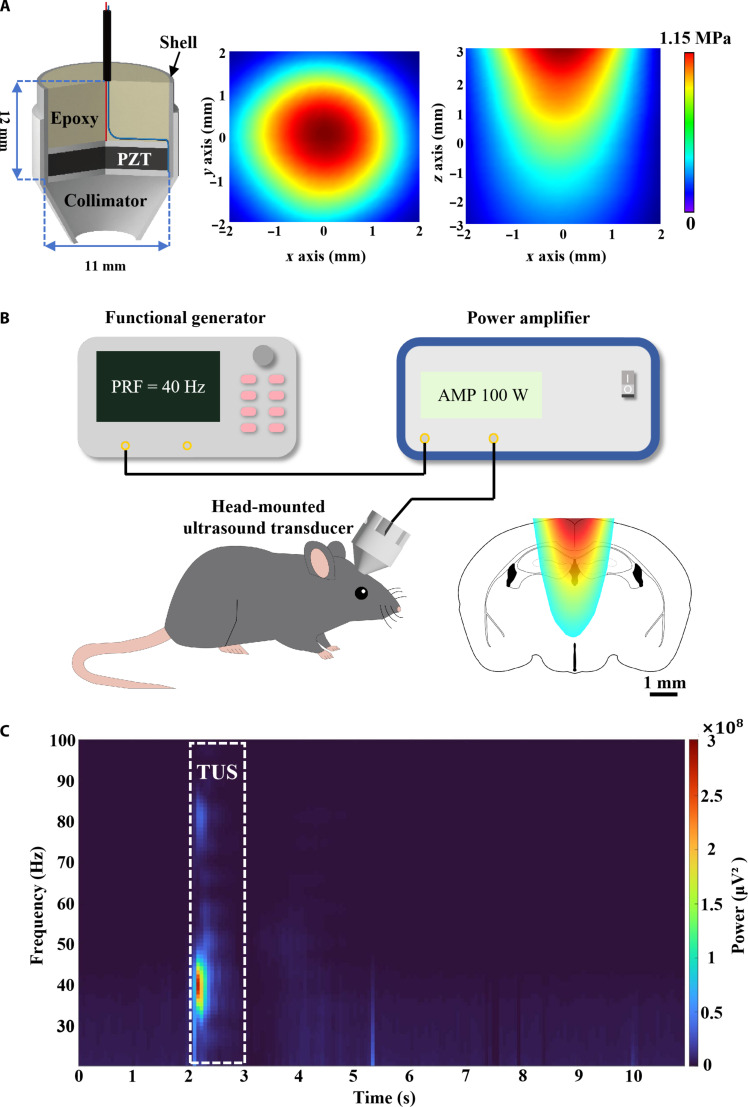
Experimental setup for transcranial ultrasound stimulation (TUS). (A) Schematic of the head-mounted ultrasound stimulation probe and distribution of the acoustic field measured in the isolated mouse skull, with the horizontal (*x*–*y*) profile on the left and the sagittal (*x*–*z*) profile on the right. The color scale indicates the normalized negative acoustic pressure of the beam, where the highest pressure is indicated in red. (B) Ultrasound stimulation device connection diagram. The ultrasound waveform is generated by a signal generator, amplified by a power amplifier, and transmitted to the ultrasound probe. (C) Spectral–temporal profile of TUS-induced brain activity at a pulse repetition frequency (PRF) of 40 Hz.

To implement TUS treatment in freely moving mice, we developed a head-mounted ultrasound transducer, characterized the acoustic field, and established a TUS treatment system specifically designed for mice (Fig. 1A and B). In wild-type (WT) mice, we conducted simultaneous stimulation-recording experiments using this ultrasound transducer while local field potentials (LFPs) were recorded in the hippocampus. The results revealed a predominant peak at 40 Hz, corresponding precisely to the ultrasound stimulation (US) PRF (Fig. 1C). Furthermore, by evaluating inter-trial phase coherence (ITPC), we demonstrated that neuronal activity consistently phase-locked to each cycle of the TUS pulsed at 40 Hz (Fig. S2e).

**Fig. 2.**
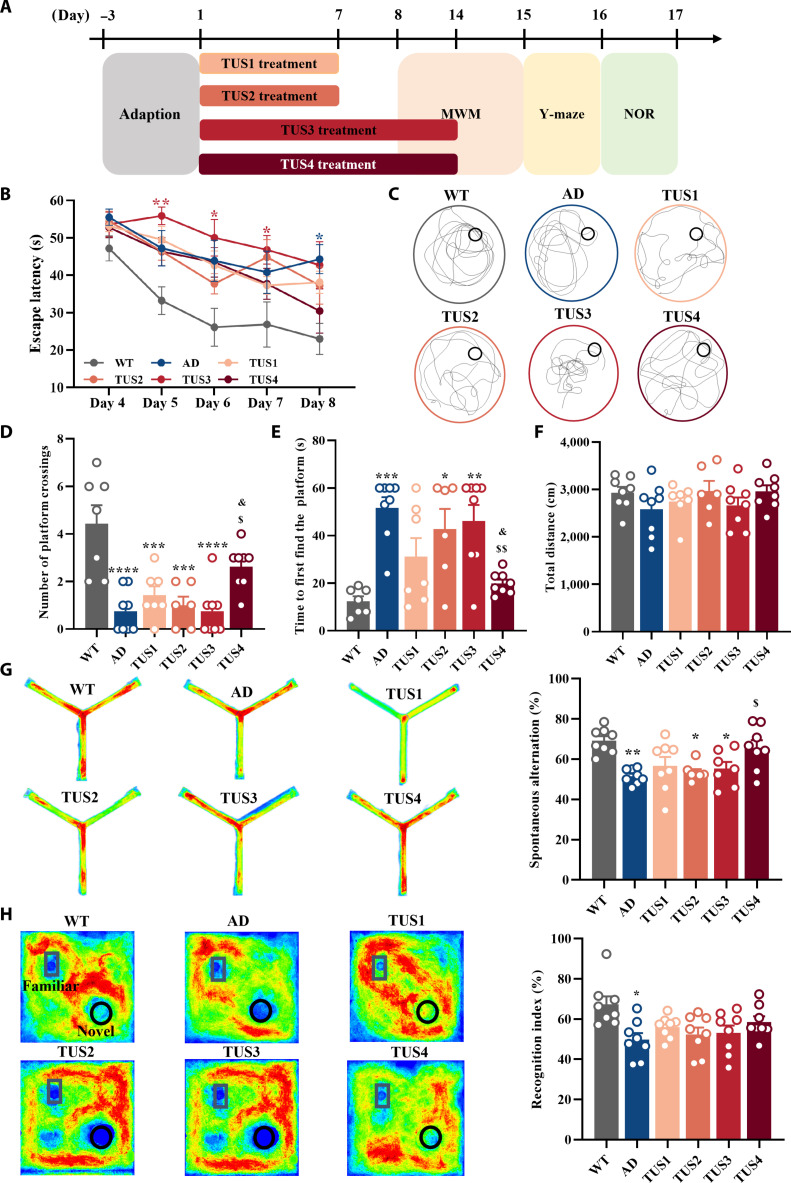
Transcranial ultrasound stimulation (TUS) attenuates the cognitive impairment of Alzheimer’s disease (AD) mice. (A) Experimental time flow diagram. (B) The escape latency of mice in different groups during the spatial learning period in the Morris water maze (MWM). (C) Typical swimming trajectories of mice in different groups during the spatial exploration period in the MWM. Quantitative analysis of the number of platform crossings (D), the time to first find the platform (E), and the total swimming distance (F) during the spatial exploration period in the MWM. (G) Typical exploration heat map and quantitative analysis of spontaneous alternation of different groups in the Y-maze test. (H) Typical exploration heat map and quantitative analysis of the recognition index in the test period of different groups in novel object recognition (NOR). *P < 0.05, **P < 0. 01, ***P < 0.001, and ****P < 0.0001 indicate statistical significance compared to the wild-type (WT) group. ^$^*P* < 0.05 and ^$$^*P* < 0.01 indicate statistical significance compared to the AD group. ^&^*P* < 0.05 indicate statistical significance compared to the TUS3 group.

The Morris water maze (MWM), the Y-maze test, and novel object recognition (NOR) were used to evaluate the effects of TUS with varying treatment durations and peak temporal-average intensity (*I*_spta_) on cognitive impairment in AD. The chart of the experiment is presented in Fig. 2A. During the spatial learning period of the MWM test, AD mice exhibited significantly increased escape latency. However, TUS1, TUS2, and TUS3 treatments failed to shorten escape latency, and any modest effect of TUS4 treatment on spatial learning did not translate into a statistically significant improvement in escape latency (Fig. 2B). Subsequently, the escape platform was removed on day 8 posttraining to complete the spatial exploration test. Typical swimming trajectories of the different groups are displayed in Fig. 2C. The AD mice group revealed a reduced number of platform crossings and increased time to first find the platform compared to the WT group; however, only the TUS4 treatment significantly reversed these changes (Fig. 2D and E). It is suggested that this parameter of TUS enhances spatial memory in AD mice. The difference in total swimming distance between the groups was statistically nonsignificant (Fig. 2F), indicating no motor ability change in this test.

In the Y-maze test, TUS-treated mice exhibited a higher spontaneous alternation rate, with the TUS4 group showing a significant increase compared to the untreated AD group (Fig. 2G) while having no effect on WT mice (Fig. S1b). In the NOR test, the recognition index (RI) was significantly lower in the AD group compared to that in WT controls. Although the TUS4-treated group exhibited a numerically higher RI than the untreated AD group, this difference did not reach statistical significance (Fig. 2H). Moreover, TUS4 did not enhance the RI in WT mice (Fig. S1c). During TUS4 treatment, the skull temperature increase remained within 1 to 2 °C (Fig. S3a). Additionally, hematoxylin and eosin (HE) staining revealed no detectable hemorrhage in the cortex or hippocampus following TUS4 treatment compared to the sham group (Fig. S3b), and Nissl staining confirmed the absence of neuronal damage (Fig. S3c). Conclusively, these results demonstrate that TUS rescues cognitive deficits in AD mice, with TUS4 treatment providing the most pronounced improvement while exhibiting a favorable safety profile. Accordingly, TUS4 was identified as the optimal parameter.

### TUS modulates neural oscillations in the hippocampal CA1 of 5xFAD mice

**Fig. 3.**
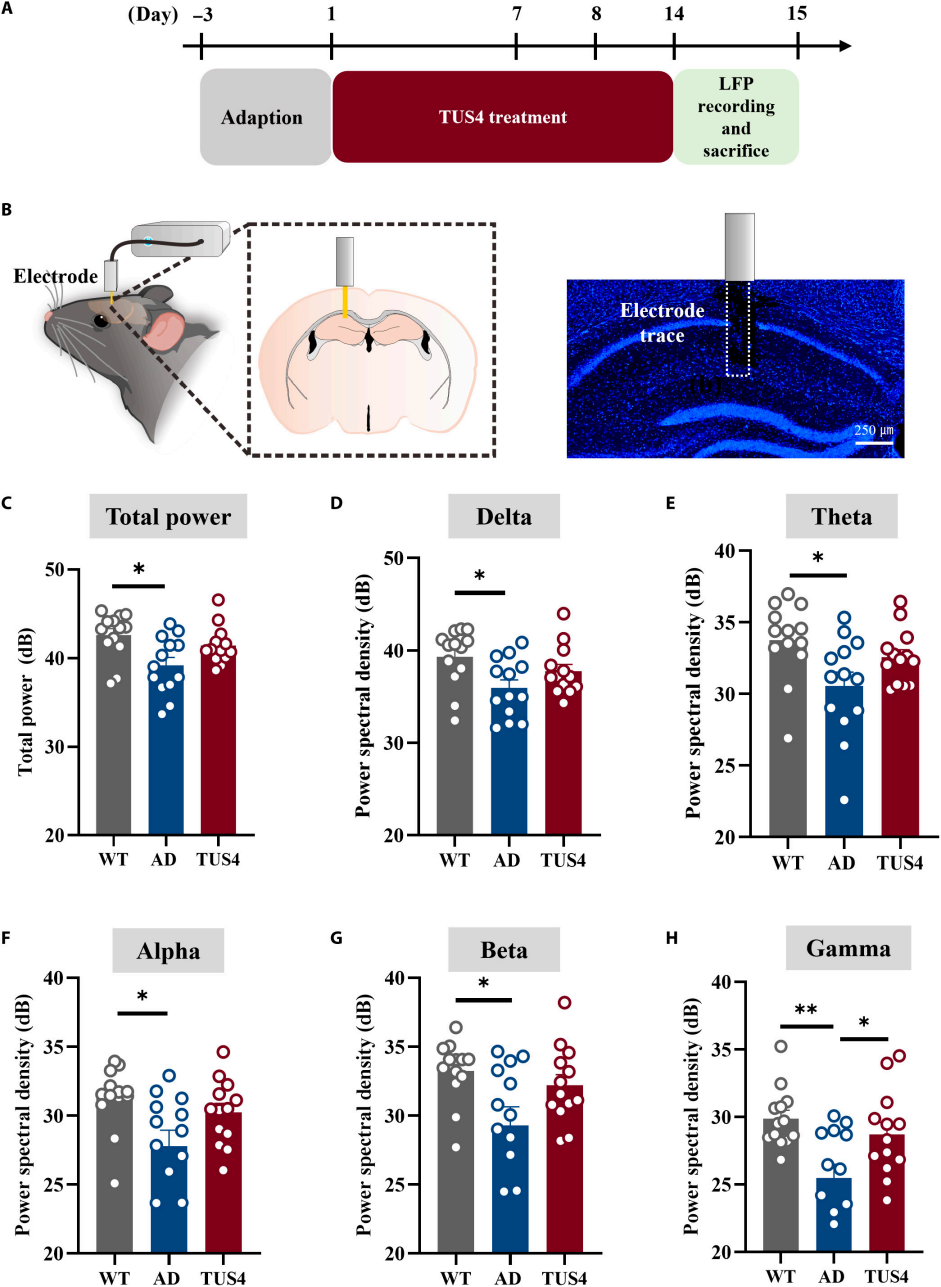
Transcranial ultrasound stimulation (TUS) alters hippocampal local field potential (LFP). (A) Experimental time flow diagram. (B) Schematics of LFP signal recording in hippocampus CA1 with histological verification of electrode placement. (C to H) Quantitative analysis of average power change in total power, delta (1 to 4 Hz), theta (4 to 8 Hz), alpha (8 to 13 Hz), beta (13 to 30 Hz), and gamma (30 to 70 Hz) bands in WT, AD, and TUS4 treatment mice. *P < 0.05; **P < 0. 01.

The synchronized rhythmic activity of neuronal assemblies, known as neural oscillations, serves to orchestrate information flow across neural circuits [24]. Variable neural oscillations in patients with AD and animal models are strongly linked to cognitive impairment, and several brain stimuli have been exhibited to alter neural oscillations [25]. Therefore, neural oscillations were further analyzed in the hippocampus through LFP assessment. The LFP power of the AD group was markedly reduced in all frequency bands relative to that of the WT group (Fig. 3C to H). This deficit was selectively rescued in the gamma band by TUS4 treatment, which elevated power to WT levels (Fig. 3H). In contrast, TUS4 treatment did not alter gamma-band power in WT mice (Fig. S1d). Furthermore, the selective restoration of gamma power was correlated with improved behavioral performance in the Y-maze test (Fig. S2a). We next evaluated cross-frequency phase–amplitude coupling (PAC) in the hippocampus. While AD mice showed impairments in both theta–gamma and delta–gamma PAC compared to WT controls, TUS4 treatment did not significantly rescue these abnormalities (Fig. S2c and d). These results demonstrate that TUS pulsed at 40 Hz significantly increased hippocampal gamma-band oscillations in AD mice. This enhancement of neural synchrony may help restore coordinated network activity, thereby providing a potential mechanism for the alleviation of cognitive impairment.

### TUS decreases Aβ levels in the hippocampus and cortex of 5xFAD mice

**Fig. 4.**
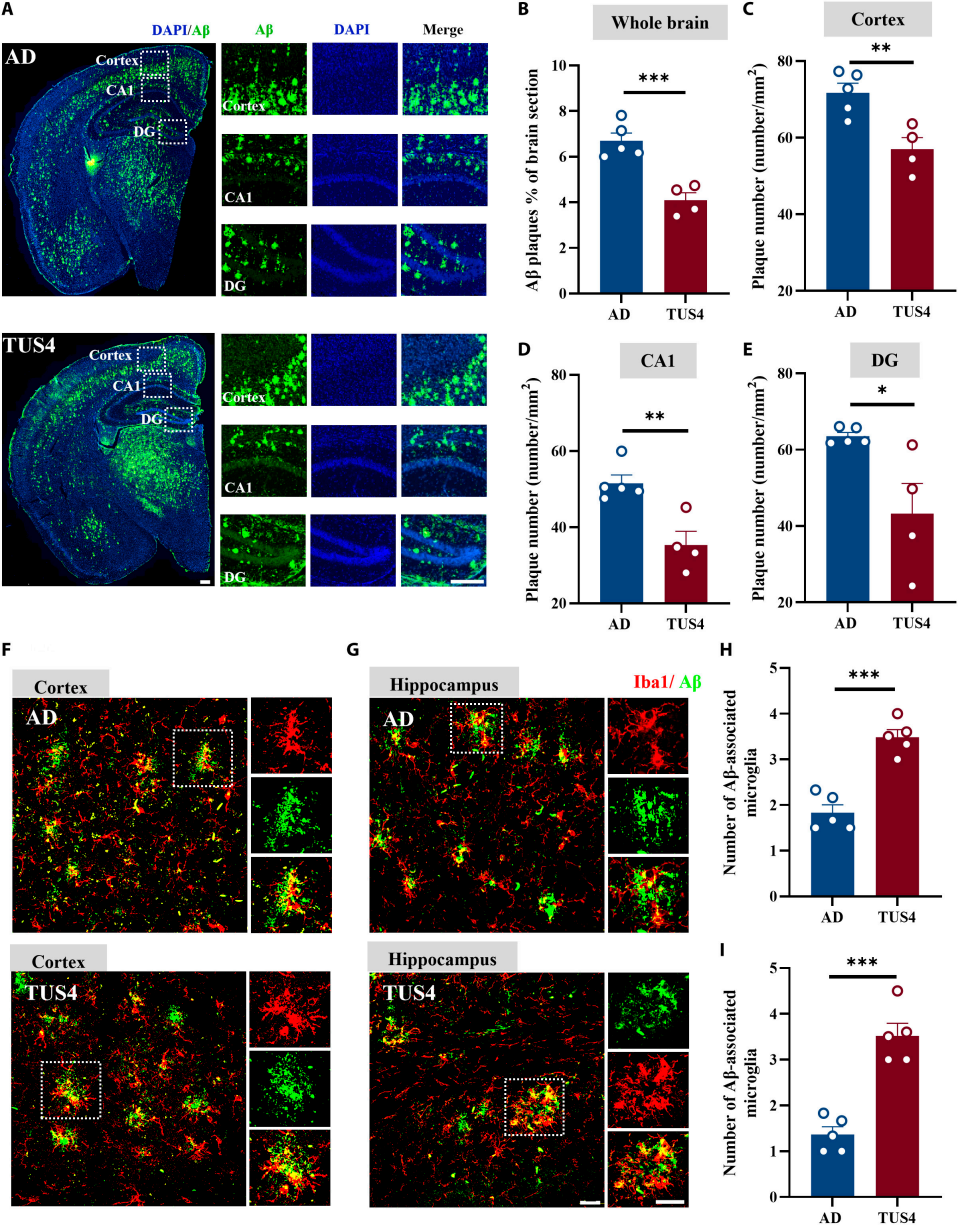
Transcranial ultrasound stimulation (TUS) decreases the Aβ level and boosts microglia responses to Aβ plaques in 5xFAD mice. (A) Representative immunofluorescence staining image of Aβ deposition in the brain sections from 5xFAD and TUS-treated 5xFAD mice. Scale bar, 250 μm. (B) Statistical analysis of the Aβ deposition of the whole brain in different groups. (C) Statistical analysis of the Aβ plaque density of the cortex in different groups. (D) Statistical analysis of Aβ plaque density of hippocampal CA1 in different groups. (E) Statistical analysis of Aβ plaque density of hippocampal DG in different groups. (F and G) Representative immunofluorescence staining images of Aβ plaque–microglia colocalization in the cortex (F) and hippocampus (G) across experimental groups. Scale bar, 25 μm. (H and I) Quantification of the number of Aβ-associated microglia in the cortex (H) and hippocampus (I). *P < 0.05; **P < 0. 01; ***P < 0.001.

Abnormal accumulation of Aβ is one of the main pathological characteristics of AD. Immunofluorescent staining was used to assess Aβ deposition in the hippocampus and cortex of AD mice. The results indicated that the increased Aβ burden in the brain of 5xFAD mice was notably reduced by TUS. This was demonstrated by a significant reduction in whole-brain Aβ levels following TUS4 treatment compared those in to the AD group (Fig. 4A and B) and a substantial decrease in Aβ density in the cortex (Fig. 4C), hippocampal CA1 region (Fig. 4D), and dentate gyrus region (Fig. 4E). Microglia, the resident immune cells in the brain, can phagocytose Aβ proteins to reduce AD pathology upon activation. To evaluate the effect of TUS treatment on Aβ phagocytosis by microglia, Aβ (green) and microglia (*Iba1*, red) were labeled using immunofluorescence staining. In both cortical and hippocampal regions, TUS4 treatment significantly increased the number of microglia surrounding Aβ plaques in AD mice (Fig. 4F to I). Furthermore, RNA sequencing (RNA-seq) analysis revealed that the expression of key genes involved in the amyloidogenic pathway, including the rate-limiting enzymes BACE1 (beta-site amyloid precursor protein cleaving enzyme 1) and PSEN1 (presenilin 1), was not significantly affected by TUS4 intervention (Fig. S4). Importantly, in WT mice, TUS4 treatment did not induce detectable increases in apoptotic cells (Fig. S5a) or in the expression of glial activation markers, such as *Iba1* and GFAP (glial fibrillary acidic protein) (Fig. S5b and c). Collectively, these findings demonstrate that TUS facilitates Aβ clearance by enhancing microglial phagocytosis without inducing overt neurotoxicity or glial inflammation, thereby contributing to reduced plaque deposition.

### TUS inhibits *RIPK1*/*NF-κB* and necroptosis in 5xFAD mice

**Fig. 5.**
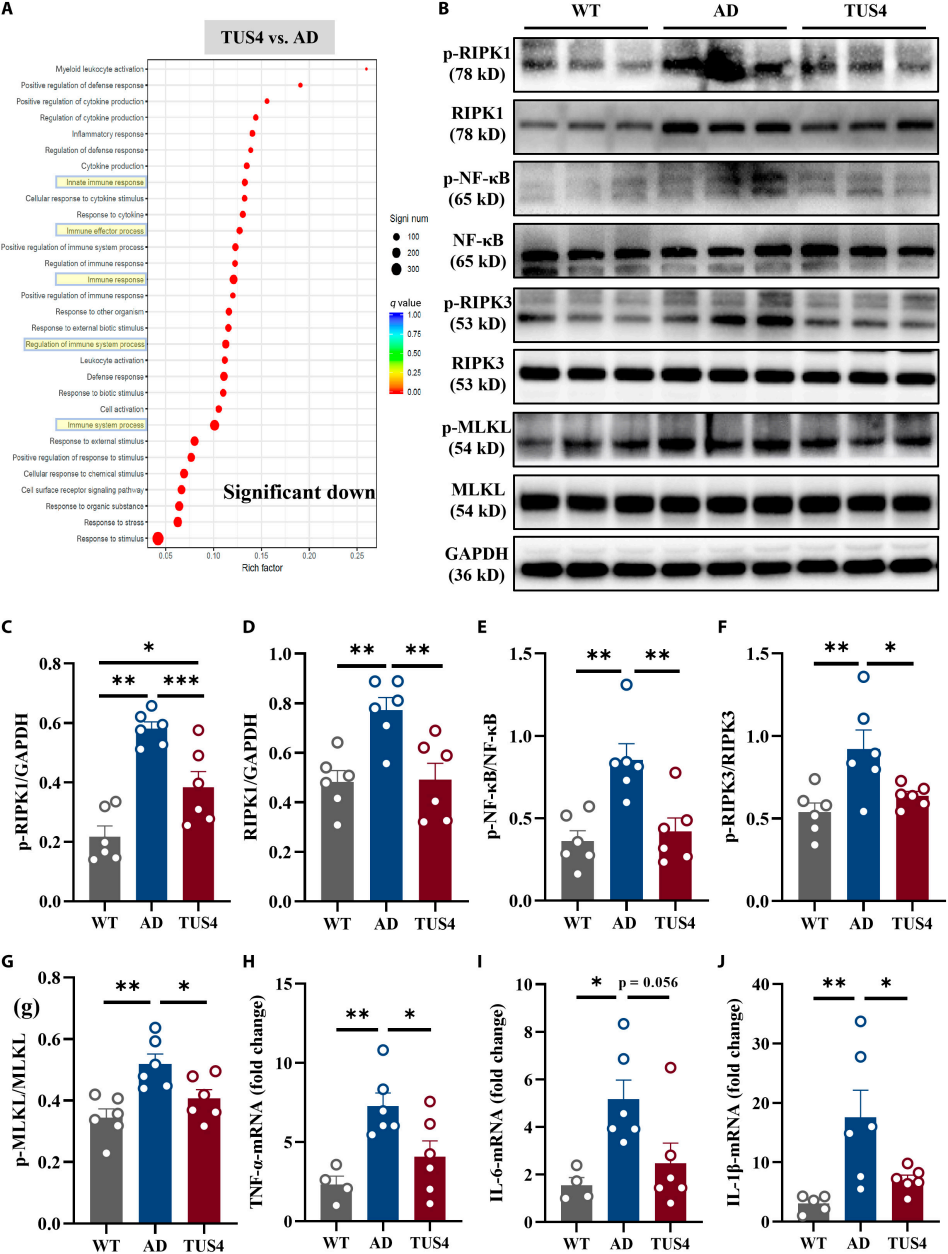
Transcranial ultrasound stimulation (TUS) attenuates neuroinflammation in 5xFAD mice by inhibiting nuclear factor kappa-B (NF-κB) and necrotic apoptosis via receptor interacting serine/threonine kinase 1 (RIPK1). (A) The Gene Ontology (GO) enrichment analysis of the differentially expressed genes in the TUS4 group vs. the AD group; the most significant 30 terms of biological process were selected to draw a scatter diagram for display. (B) Representative bands in the hippocampus of mice in different groups. Glyceraldehyde-3-phosphate dehydrogenase (GAPDH) was used as a loading control. Quantitative analysis of p-RIPK1 (C), RIPK1 (D), p-NF-κB (E), p-RIPK3 (F), and p-MLKL (G) protein expression. (H to J) The messenger RNA (mRNA) levels of the genes of tumor necrosis factor-α (TNF-α), interleukin-6 (IL-6), and interleukin-1β (IL-1β) in the hippocampus of mice in different groups. *P < 0.05; **P < 0. 01; ***P < 0.001.

To clarify the underlying mechanisms by which TUS improves cognitive impairment in AD, mouse hippocampal tissues were isolated for comprehensive bioinformatics analysis via transcriptome-wide RNA-seq. A total of 1,922 differentially expressed genes (DEGs) were identified in AD mice relative to WT controls, with 1,522 up-regulated and 400 down-regulated. Additionally, comparative analysis with the AD group identified 273 up-regulated and 721 down-regulated DEGs following TUS4 treatment (Fig. S6a). In Gene Ontology (GO) enrichment analysis, the top 30 biological process functions were identified, with the results indicating that after TUS4 treatment, most biological-process-related genes were enriched in the innate immune response, immune response, and immune system process and were significantly down-regulated (Fig. 5A). Kyoto Encyclopedia of Genes and Genomes (KEGG) analysis further revealed changes in the *NF-κB* pathway and other related pathways following TUS4 treatment (Fig. S6c).

*RIPK1* serves as a pivotal mediator of neuroinflammation in neurodegenerative diseases, promoting inflammatory cytokine production through *NF-κB* pathway activation [26,27]. Furthermore, *RIPK1* can trigger the apoptosis of necrotic cells by activating *RIPK3*, leading to necrotic-like changes and the release of inflammatory factors [28]. Consequently, the effect of TUS on these processes was further evaluated. RNA-seq results revealed that the *RIPK1* gene was significantly down-regulated following TUS4 treatment compared to that of the AD group (Fig. S6d), while the same results were obtained in reverse transcription quantitative polymerase chain reaction (RT-qPCR; Fig. S6e). Western blotting revealed that the expression levels of phosphorylated *RIPK1*, *RIPK1*, and phosphorylated *NF-κB* proteins in the hippocampus of AD mice was significantly elevated compared to those of WT mice, while TUS4 treatment significantly reduced the expression of these proteins (Fig. 5C to E). Consistent with the reduction in *RIPK1* activation, the levels of hippocampal necroptosis markers, notably phosphorylated *RIPK3* and *MLKL*, were also significantly reduced by TUS4 (Fig. 5F and G). The anti-inflammatory effects of TUS4 were further confirmed by RT-qPCR analysis of hippocampal tissues, which showed significant reductions in the messenger RNA levels of *TNF-α* (Fig. 5H), *IL-6* (Fig. 5I), and *IL-1β* (Fig. 5J).

### US reduces the inflammatory response and necroptosis induced by Aβ in BV2 cells

**Fig. 6.**
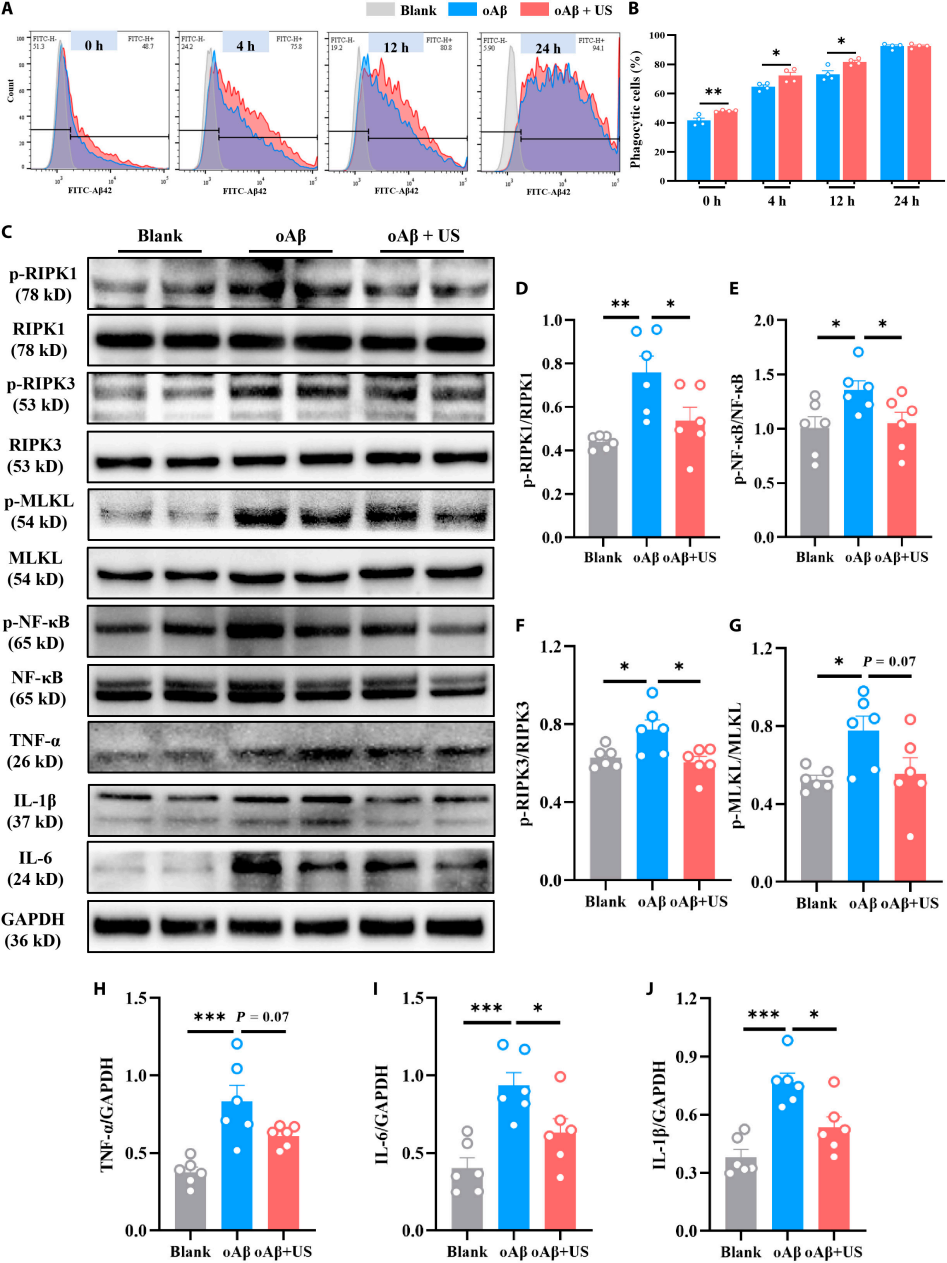
Ultrasound stimulation (US) alleviated Aβ-induced inflammatory responses by inhibiting receptor interacting serine/threonine kinase 1 (RIPK1) in BV2 cells. (A) Comparison of the percentages of the numbers of phagocytosed Aβ-positive cells in the US and oligomer Aβ (oAβ) groups at different time points. The gray histogram represents blank control. (B) Quantification of phagocytosed Aβ-positive cells in different groups. (C) Representative bands in the hippocampus of mice in different groups. Glyceraldehyde-3-phosphate dehydrogenase (GAPDH) was used as a loading control. Quantitative analysis of p-RIPK1 (D), p-NF-κB (E), p-RIPK3 (F), p-MLKL (G), tumor necrosis factor-α (TNF-α) (H), interleukin-6 (IL-6) (I), and interleukin-1β (IL-1β) (J) protein expression. *P < 0.05; **P < 0. 01; ***P < 0.001.

*RIPK1* was highly expressed in microglia from patients with AD and mice [29]. Consistently, we observed that *RIPK1* expression was predominantly elevated in microglia within the brain of AD mice (Fig. S7). To further investigate whether US has a positive therapeutic effect on AD by modulating *RIPK1* in microglia, in vitro experiments were conducted using BV2 cells for relevant validation. We first characterized the acoustic field of the ultrasound transmitted through the cell culture plate to confirm that cultured cells were effectively exposed to the stimulation (Fig. S8a and b). We found that US did not significantly affect BV2 cell viability or *RIPK1* expression (Fig. S8c and d). Subsequently, BV2 cells were incubated with Alexa Fluor 488-labeled Aβ, and the endocytosis of Aβ by BV2 cells was analyzed by flow cytometry at the indicated time points. The results demonstrated that BV2 cells could phagocytose Aβ, and US further accelerated this process until BV2 reached saturation at 24 h (Fig. 6A and B). The expression of target proteins in microglia was assessed by western blotting. The results revealed that US decreased the expression of phosphorylated *RIPK1* (Fig. 6D), phosphorylated *NF-κB* (Fig. 6E), phosphorylated *RIPK3* (Fig. 6F), and phosphorylated *MLKL* (Fig. 6G) and also reduced the expression of inflammatory factors including *TNF-α* (Fig. 6H), *IL-6* (Fig. 6I), and *IL-1β* (Fig. 6J). In contrast, in the absence of Aβ stimulation, US treatment alone did not alter the expression of these inflammatory cytokines in BV2 cells (Fig. S8e). Consequently, both in vitro and in vivo data showed that US promoted Aβ phagocytosis by microglia and reduced the inflammatory factors’ release. These changes were accompanied by suppressed *NF-κB* signaling and reduced *RIPK1*-mediated necroptosis, which correlated with the observed functional improvements.

### TUS increases the density of synaptic dendritic spines in the hippocampus

**Fig. 7.**
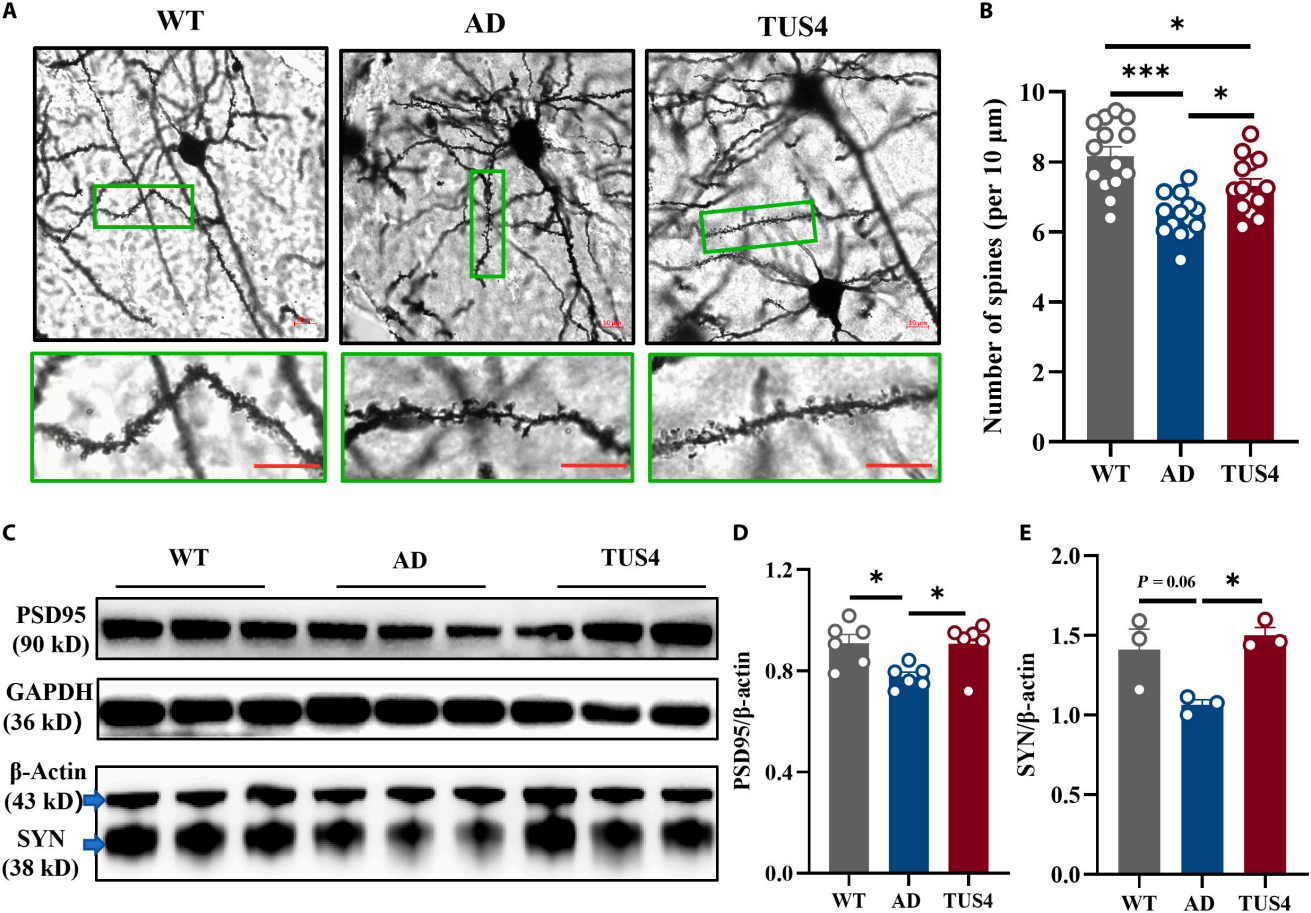
Transcranial ultrasound stimulation (TUS) increases the synaptic plasticity of the hippocampus in AD mice. (A) Representative images of dendritic spines in the hippocampus of mice in different groups. Scale bars: 10 μm. (B) Quantification of dendritic spine density in the hippocampus. (C) Representative bands in the hippocampus of mice in different groups. β-Actin and glyceraldehyde-3-phosphate dehydrogenase (GAPDH) were used as loading controls. (D-E) Quantification of PSD-95 and SYN protein expression. *P < 0.05; ***P < 0.001.

Synaptic plasticity in the hippocampus plays a vital role in cognitive functions in mice [30]. GO enrichment analysis of RNA-seq highlighted a pronounced up-regulation of genes associated with synaptic signaling (Fig. S6b). Subsequently, Golgi staining was used to assess the density of dendritic spines in the hippocampus. The results demonstrated that TUS4 treatment produced significant main effects on spine density. Hippocampal dendritic spine density was significantly reduced in AD mice compared to that in WT controls. In contrast, TUS4 treatment effectively rescued this deficit (Fig. 7A and B). Furthermore, western blotting was utilized to detect *PSD95* and *SYN*, which regulate synaptic activity and plasticity. Quantification of hippocampal synaptic proteins revealed significantly lower expression levels of *PSD95* and *SYN* in AD mice relative to those in WT mice. Treatment with TUS4 increased hippocampal synaptic proteins in AD mice (Fig. 7C to E).

## Discussion

This study developed a head-mounted planar transducer that allows mice to undergo TUS pulsed at 40 Hz while engaging in free movement. TUS parameters were optimized by adjusting *I*_spta_ and the total intervention duration, finding that a continuous intervention at 2.14 W/cm^2^ for 14 d significantly improved cognitive deficits, reduced intracerebral Aβ plaques, and enhanced neural oscillations in AD mice. Furthermore, investigation indicated that TUS intervention inhibited the *RIPK1*/*NF-κB* pathway and necroptosis, decreased neuroinflammation in AD, and increased synaptic plasticity in the hippocampus. These findings represent a novel preclinical outcome, suggesting a potential therapeutic approach for AD.

Brain stimulation techniques have emerged as a crucial complementary tool for intervening in neurodegenerative diseases. TUS offers enhanced spatial selectivity compared to transcranial electrical stimulation and transcranial magnetic stimulation, which is essential for modulating deep brain regions. The hippocampus is a deep brain region closely related to cognition, and a study demonstrated that the US of the unilateral hippocampus can improve cognitive deficits and decrease Aβ plaque accumulation in AD mice [22]. Given the widespread nature of Aβ deposition in AD, we developed a head-mounted planar transducer to deliver US across a larger brain volume (Fig. 1A), encompassing bilateral hippocampal and cortical regions. This design enables TUS application in freely moving mice without the confounding effects of anesthesia, thereby aligning the intervention with the diffuse pathology of the disease. It is established that anesthetics can exacerbate AD pathology [31,32]. Administering TUS under anesthesia may therefore compromise its efficacy and prolong neural response latency [33]. Consequently, we implemented TUS in awake, freely moving mouse. Beyond avoiding anesthetic confounds, this approach—particularly when integrated with wearable technology—holds strong translational relevance, thereby enhancing its potential for the long-term clinical management of brain disorders. However, translating findings from animal models to human applications necessitates careful consideration of anatomical differences, such as skull thickness and brain volume, which critically affect transcranial ultrasound penetration, focusing, and ultimately the acoustic dose delivered to the target region. Therefore, systematic parameter optimization for human applications may be required, including the use of lower fundamental frequencies to reduce cranial attenuation, the adoption of multielement phased arrays to correct phase distortion and energy loss after transcranial propagation, and appropriate extension of treatment duration to ensure sufficient acoustic energy delivery. A thorough reassessment of safety profiles under these adjusted parameters is imperative.

Gamma stimulation is considered a compelling approach to modifying AD progression [34]. A foundation for gamma entrainment in AD was laid by Tsai and colleagues [16,35], who showed that 40-Hz visual stimulation reduces amyloid plaques and engages neuroprotective responses. Crucially, the therapeutic potential of gamma entrainment extends beyond sensory modalities, as 40-Hz electrical and magnetic stimulation also ameliorate AD pathology and cognition without involving visual or auditory mechanisms [19,36]. However, these techniques are limited in their ability to noninvasively target deep brain structures. This limitation highlights the unique advantage of TUS, which can precisely focus energy on deep memory-related regions [21]. Building on the pioneering work of Bobola et al. [22], which established that short-term TUS pulsed at 40 Hz activates microglia and reduces amyloid burden. our study extends these findings within a more translationally relevant framework. Unlike prior protocols often limited by anesthesia or focal targeting, we employed a wearable ultrasound transducer to administer chronic stimulation over broader brain regions—including the bilateral hippocampus and cortex—in freely behaving AD mice. This sustained paradigm not only corroborated the cognitive and amyloid-clearing benefits of TUS pulsed at 40 Hz (Fig. 2) but further revealed, through parameter optimization, that therapeutic efficacy depends on both elevated spatial-peak temporal-average intensity (*I*_spta_) and extended treatment duration. This energy- and intervention-time-dependent response aligns with the progressive pathology of AD and underscores the importance of developing clinically feasible, long-term TUS regimens. Furthermore, TUS specifically enhanced gamma oscillation power in the hippocampus without affecting neural activity in other frequency bands (Fig. 3), and this enhancement was positively correlated with improved cognitive performance in the Y-maze test (Fig. S2a). The consistent and persistent 40-Hz input may induce strong rhythmic neuronal firing that spreads across the brain, enhancing neuronal oscillations within the gamma frequency band. This enhancement may facilitate the recovery of cognitive deficits in AD and further support the positive role of frequency-specific entrainment stimulation in modulating brain function [24,34]. Consistent with this notion, our simultaneous stimulation-recording experiments in WT mice demonstrated that TUS pulsed at 40 Hz reliably entrained hippocampal neurons (Fig. 1C) and significant phase locking of neuronal activity to each stimulation cycle (Fig. S2e), providing direct evidence for the entrainment of gamma rhythms by TUS pulsed at 40 Hz. Future studies should further validate whether this entrainment effect occurs in AD models and explore its potential propagation to other brain regions involved in cognition. Furthermore, under the effective TUS parameters, intracranial temperature monitoring showed only a mild elevation (1 to 2 °C; Fig. S3a). Histologically, no evidence of cerebral hemorrhage or neuronal damage was observed (Fig. S3b and c). Additionally, there were no detectable increases in apoptotic cells (Fig. S5a) or in the expression of glial activation markers (Fig. S5b and c). Importantly, in WT mice, the same TUS protocol did not alter cognitive performance or gamma-band oscillations (Fig. S1d), suggesting that its beneficial effects in AD models are not attributable to generalized brain excitation or nonspecific stimulation but likely reflect a targeted restoration of impaired neural and glial function. Collectively, these data substantiate the safety of the stimulation paradigm and support its disease-modifying potential in the absence of overt tissue injury or off-target neural modulation.

Microglia serve as the principal immune cells of the central nervous system, maintaining brain homeostasis by exerting immunosurveillance and clearance of misfolded proteins, pathogens, and cellular debris [37]. In AD, resident resting microglia are activated and migrate to regions of Aβ plaques to facilitate their clearance. TUS has been demonstrated to enhance the phagocytic activity of microglia to clear Aβ deposits [22,38]. Corroborating these findings, our in vivo and in vitro results revealed a significantly higher ratio of recruited microglia to Aβ plaque phagocytosis within the US-treated group (Figs. 4 and 6), suggesting an enhanced capacity for Aβ clearance. Importantly, TUS intervention did not significantly alter the expression of key genes in the amyloidogenic pathway, including the rate-limiting enzymes BACE1 and PSEN1 (Fig. S4). These results indicate that the observed plaque reduction was driven primarily by enhanced clearance mechanisms rather than suppressed production. Additionally, microglia are mechanosensitive cells capable of responding to alterations in the extracellular mechanical milieu [39]. Previous studies indicate that tissue stiffness is elevated in Aβ plaque-associated regions, accompanied by a selective up-regulation of the mechanosensitive ion channel Piezo1 in associated microglia [40]. Acoustic radiation force generated by ultrasound has been demonstrated to modulate the activity of such mechanosensitive channels [41]. Furthermore, research suggests that ultrasound may directly excite neurons by inducing nanoscale cavitation events within the lipid bilayer of the cell membrane, even in the absence of microbubbles [42]. In the present study, the mechanical index (MI) of the ultrasound applied was maintained below 1.9, a threshold considered sufficient to avoid significant inertial cavitation [43]. Given the only mild intracranial temperature rise observed in our experiments (Fig. S3a), we propose that the primary bio-effective component of TUS under our conditions likely stems from its nonthermal mechanical force—specifically, acoustic radiation force—rather than from thermal or cavitation effects. This acoustic force may directly activate mechanosensitive molecules such as Piezo1 on the microglial membrane, thereby initiating downstream intracellular signaling that ultimately drives morphological remodeling, immune activation, and enhanced phagocytic function. Moreover, chronic activation of microglia results in the release of pro-inflammatory cytokines and chemokines that induce neuroinflammation, thereby exacerbating AD progression [44]. *RIPK1* is a multidomain serine/threonine kinase characterized by an N-terminal kinase domain, a C-terminal death domain, and an intermediary domain connecting the 2 [45,46]. Immunofluorescence staining of human AD postmortem cortical samples revealed a significant increase in *RIPK1*, which positively correlated with the pathological progression of the disease [47]. Moreover, elevated levels of *RIPK1* were observed in AD mouse models, with prominent expression in microglia in both murine and human brains [29]. These findings were confirmed in this study (Figs. S6 and S7). A previous study revealed that *RIPK1* activation is crucial in microglia-mediated neuroinflammation linked to neurological disorders [48]. Within *TNF-α* signaling, *RIPK1* amplifies the inflammatory response by assembling a pivotal complex with partners like tumor necrosis factor receptor-associated death domain protein (TRADD) and tumor necrosis factor receptor associated factor 2 (TRAF2). This assembly is essential for *NF-κB* pathway activation, which in turn drives the production of pro-inflammatory cytokines [26,27]. Given its role in driving neuroinflammatory and degenerative processes, *RIPK1* therefore emerges as a compelling target for AD intervention. Inhibition of *RIPK1* through pharmacological and genetic means has been confirmed to lower Aβ plaque and inflammatory cytokine within APP/PS1 mice, resulting in improved memory deficits. Furthermore, *RIPK1* inhibition could facilitate the breakdown of Aβ by microglia in vitro [29]. Our findings indicated that TUS significantly down-regulated both *RIPK1* and phosphorylated *RIPK1* in the hippocampus of AD mice, thereby inhibiting the *NF-κB* signaling pathway, which likely constitutes the alleviation of neuroinflammation (Fig. 5 and Fig. S6). Additionally, in our in vitro study, the regulatory effect of TUS on *RIPK1* in microglia was identified (Fig. 6). Importantly, this effect appears to be context dependent. As shown in Fig. S8, the same US applied to BV2 cells in the absence of Aβ did not significantly alter *RIPK1* expression or the release of key inflammatory cytokines. This contrast suggests that TUS does not intrinsically modulate microglial inflammatory signaling, but rather, its regulatory action on the *RIPK1*/*NF-κB* pathway is specifically engaged within the AD pathological milieu, likely in response to Aβ burden. Moreover, *RIPK1*-induced necroptosis has been demonstrated to activate immune cells and enhance inflammatory responses [49]. Its inhibition has been found to reduce neuroinflammation and improve both pathological and cognitive functions in AD [29,50]. Our integrated in vivo and in vitro findings revealed the inhibitory effect of TUS on necrotic apoptotic proteins (Figs. 5 and 6), which may contribute to the alleviation of neuroinflammation in AD and support the recovery of cognitive function.

Despite the promising findings, this study has some limitations that should be addressed. First, to mitigate the confounding effects of sex on AD pathology, this study was deliberately conducted in female 5xFAD mice. While this approach enhances internal validity, it necessarily delimits the generalizability of our conclusions. Future investigations should therefore incorporate both male subjects and diverse genetic backgrounds to substantiate the broader applicability of TUS. Second, while parameter optimization was performed in this study, a detailed investigation of *I*_spta_ and intervention duration was not conducted. This scope was defined primarily by safety considerations and technical feasibility, as increasing *I*_spta_ would elevate the MI, thereby introducing risks of adverse bioeffects such as cavitation and tissue damage. Prolonging the intervention duration, meanwhile, presents a distinct technical challenge: ensuring stable and reliable positioning of the noninvasive head-mounted collimator in a freely behaving animal model. Beyond these immediate constraints, future work should aim to systematically optimize other programmable parameters, including duty cycle and sonication duration, which represent promising avenues for enhancing therapeutic efficacy and translational feasibility. Finally, the absence of validation through gene-edited mouse models limited the investigation of the mechanisms underlying the relationship between *RIPK1* in microglia and neuroinflammation in TUS treatment. It should be noted that the lack of genetic manipulation models means that we could not obtain definitive evidence that TUS alleviates neuroinflammation specifically by targeting *RIPK1* in microglia and the *RIPK1*/NF-κB pathway. Future studies should focus on using gene-edited mice to clarify the functional implications of *RIPK1* in microglial cells and their impact on neuroinflammatory processes. Such investigations will be crucial for understanding the therapeutic mechanisms of TUS treatment.

Conclusively, this study demonstrates that gamma entrainment efficacy by US is contingent upon precise parameter selection. The therapeutic benefits of TUS pulsed at 40 Hz likely arise from its coordinated actions on neural circuit function and neuroimmune homeostasis. Specifically, the treatment enhances gamma oscillations and synaptic integrity, while its mechanical energy may also directly engage microglia, potentially leading to the observed down-regulation of the *RIPK1*/*NF-κB* pathway and attenuation of neuroinflammation. Collectively, these parallel modifications are associated with reduced Aβ pathology and improved cognitive performance, underscoring the promising therapeutic potential of this noninvasive neuromodulation strategy.

## Materials and Methods

### Animals

C57BL/6J mice (20 to 22 g, 8 to 10 weeks of age) were sourced from the Vital River Laboratory Animal Technology (Beijing, China). 5xFAD transgenic mice were sourced from Jackson Laboratory and maintained by mating with littermate nontransgenic mice. The genotype of breeding mice was verified using polymerase chain reaction (PCR) analysis of tail DNA. To control for the known effect of sex on pathology and behavior in 5xFAD mice [51], this study utilized only female mice at 6 months of age. Mice were divided into 6 groups: nontransgenic mice as the WT group, transgenic mice as the sham TUS (AD), and different TUS groups (TUS1, TUS2, TUS3, and TUS4). The animal holding room was maintained at a stable temperature (23 to 25 °C) and humidity levels (50% to 60%). Mice were group-housed under a standard 12-h light/dark cycle with free access to food and water.

### Head-mounted transducer and TUS treatment

To deliver ultrasound waves to mice brain during free movement, lead zirconate titanate piezoelectric ceramic sheets were customized and soldered electrodes, following previously published methods [52]. These components were embedded in a 3-dimensionally printed (3D-printed) cylindrical housing (12 mm in height and 11 mm in diameter), with the remaining space filled with epoxy resin to serve as backing for making the head-mounted transducer (Fig. 1A). Ultimately, a matching conical collimator was fabricated using a 3D printer and secured to the skull of the mice. The ultrasound field and acoustic pressure were characterized using a needle hydrophone (Precision Acoustics, UK) and an automatic 3D acoustic field scanning system (Boray Technology, China). The map of the acoustic field measured with an ex vivo mouse skull is presented in Fig. 1A. By comparison with the mouse brain atlas, ultrasound can essentially cover the hippocampus bilaterally (Fig. 1B).

In the in vivo experiments, following anesthesia with isoflurane, each mouse was placed in a stereotactic apparatus for head fixation. The collimator was mounted on the skull, centered 1.58 mm behind the bregma. Mouse was allowed to recover from surgery and acclimatize to the collimator for 3 d. The fabricated ultrasound transducer was attached to the mouse head by inserting it into the collimator filled with a couplant. Ultrasound pulsation was delivered by a signal generator (RIGOL Technology, China) with subsequent amplification via a power amplifier (Electronics & Innovation, USA) and applied to the transducer, which traveled via the skull to act on the brain. The parameters for ultrasound delivery were as follows: 0.5-MHz center frequency, 40-Hz PRF, 1.25-ms tone burst duration, 1-s sonication duration, and 10 s interstimulus interval. TUS was performed twice daily for 20 min. In the parameter study, the spatial-peak temporal-average intensity (*I*_spta_: 0.33 or 2.14 W/cm^2^) and total treatment duration (7 or 14 d) were varied, including TUS1 (0.33 W/cm^2^ for 7 d), TUS2 (2.14 W/cm^2^ for 7 d), TUS3 (0.33 W/cm^2^ for 14 d), and TUS4 (2.14 W/cm^2^ for 14 d), while other parameters were kept the same. For sham treatment, the ultrasound transducer was placed on the head of mice; however, no ultrasound wave was emitted. The *I*_spta_ and total treatment duration were determined based on a previous study [22,23,53]. The selected *I*_spta_ parameter corresponded to an *I*_sppa_ of 42.6 W/cm^2^, which is well below the Food and Drug Administration 190 W/cm^2^ limit for diagnostic ultrasound [22].

Furthermore, the optimized TUS parameters were applied to WT mice to differentiate disease-specific therapeutic effects from general neuromodulatory responses and to assess the histological safety via HE and Nissl staining. C57BL/6J mice were randomly allocated into 2 experimental groups: one receiving sham TUS (sham) and the other receiving TUS with the optimized parameters. The experimental design is illustrated in Fig. S1a.

For in vitro experiments, ultrasound was applied through the transducer positioned beneath the culture plate. Prior to stimulation, the acoustic field distribution was characterized by placing a section of the culture plate bottom in front of the collimator to measure the ultrasound field after it passed through the plate (Fig. S8a). During stimulation, the following parameters were applied to the single-cell-type culture: the peak negative acoustic pressure was set to 0.33 W/cm^2^ with a total time of 10 min. The remaining parameters were the same as those in the in vivo experiment.

### Learning and memory behavioral test

#### Y-maze test

To assess transient spatial working memory, mice were tested in a Y-maze consisting of 3 horizontal arms (35 cm in length, 6 cm in width, and 10.5 cm in height) positioned at 120° angles. The detailed protocol and the calculation of the spontaneous alternation rate were performed as previously described [54]. The maze was wiped down with 70% ethanol before each trial to eliminate residual odors.

#### NOR test

The NOR test included the training and test phases. In the habituation phase, the mouse was introduced to the testing arena (50 cm *L* × 50 cm *W* × 100 cm *H*) to explore 2 identical objects for 10 min. The test session was conducted 1 h following the training session. In the same box, one familiar object was exchanged with a novel object of a different shape but a similar height. The mouse was placed again in the box for 10 min of exploring. The interior of the box was cleaned with 70% ethanol to eliminate any irrelevant odors between test turns. The exploration time directed at each object was recorded. The RI was quantified as the time spent exploring the novel object divided by the total exploration time for both objects. Active exploration in mice was defined as direct interaction with an object involving a nose within 1.5 cm and/or touching an object with the nose or tentacles. Climbing and sitting on objects was not considered exploratory behavior.

#### MWM test

The MWM apparatus test was conducted in a circular pool (120 cm in diameter) containing a submerged escape platform (8 cm in diameter). To facilitate tracking of the behavior of mice, the water was made opaque using titanium dioxide, and the water temperature was held at 20 ± 2 °C. The test lasted for 8 d, with daily procedures performed in accordance with a previously published methodology [55]. The number of platform crossings and time to first find the platform along with locomotor activity (total distance of swimming) were recorded.

### LFP recording and analysis

The mouse was anesthetized using isoflurane and secured on the stereotaxic apparatus (RWD Life Science Co., China) while a heating pad maintained the body temperature at approximately 37 °C. A modified electrode was implanted in the CA1 of the hippocampus at the following coordinates: anteroposterior = −1.94 mm, mediolateral = 1.2 mm, and dorsoventral = −1.5 mm. Two microscrews were fixed to the right frontal and left occipital bones to access the reference electrode and ground wire, respectively. The electrode leads were inserted into a 7-pin connector and connected to the recording system (Solar Electronic Technologies Co., China). The LFP signals were recorded with a sampling frequency of 1 kHz, and the recording duration was 5 min. The brain tissue was collected and subjected to immunofluorescence to identify the location of electrode insertion. The data were processed and analyzed offline using the MATLAB software (version 2023a), with the pwelch function employed to calculate the power spectral density of LFP signals. Additionally, the LFP signals were divided into standard frequency bands (delta: 1 to 4 Hz; theta: 4 to 8 Hz; alpha: 8 to 13 Hz; beta: 13 to 30 Hz; gamma: 30 to 70 Hz). PAC was quantified using the Tort modulation index, which is based on the Kullback–Leibler divergence between the observed distribution of high-frequency amplitude relative to low-frequency phase and a uniform distribution [56]. The amplitude and phase components of the signals were extracted via the Hilbert transform. Given the technical challenges associated with integrating a wearable US transducer with a chronic in vivo LFP-recording system, simultaneous stimulation-and-recording experiments were performed in WT mice to analyze ITPC. Mice were anesthetized and fixed in a stereotaxic frame. Electrode wires implanted in the hippocampus were embedded into grooves that had been preground on the skull without penetrating it. A collimator was then secured to the skull, and the US transducer was placed inside the collimator. Anesthesia was discontinued, allowing the mice to move freely and acclimate for 1 h. LFP signals were first recorded from living mice under TUS. Immediately thereafter, the mice were euthanized, and LFP signals were again recorded under the same TUS condition. The time–frequency representation of LFP from living mice was subtracted from that obtained from dead mice under the corresponding condition, thereby removing the 40-Hz electromagnetic pulse artifact generated by the transducer. For ITPC analysis, the 40-Hz component was first extracted via band-pass filtering. Each recording session was then segmented into trials aligned with the stimulation period. The Hilbert transform was applied to each trial to obtain the instantaneous phase at each time point. For each time point, the mean vector length of the instantaneous phases across trials was calculated as the ITPC value. The resultant vector length was taken as the ITPC value. ITPC was calculated according to the following formula:

$$\text{ITPC}=\left|\frac{1}{N}\sum_{K=1}^{N}e^{i\theta_{k}}\right|$$ (1)

### Assessment of skull temperature during TUS

To determine whether TUS application results in skull temperature elevation, mice were positioned in a stereotaxic frame and subjected to treatment. Throughout the procedure, skull temperature was continuously monitored using an infrared thermal imaging camera (NEC AVIO, Japan).

### Histopathological staining

#### Immunofluorescence staining

Mouse brain sections (30 μm) were prepared and subjected to immunofluorescence staining as previously described [57]. The following primary antibodies were used: anti-Aβ (1:2,000, BioLegend), anti-*Iba1* (1:1,000, Fujifilm), anti-*RIPK1* (1:500, ABclonal), and anti-GFAP (1:1,000, Invitrogen). Corresponding secondary antibodies (donkey anti-rabbit Alexa Fluor 555/488, goat anti-mouse Alexa Fluor 488) were used at 1:1,000. Nuclei were counterstained with 4′,6-diamidino-2-phenylindole (Beyotime, China) for 10 min at room temperature. Brain sections were subsequently mounted using an antifade mounting medium (Beyotime, China) and examined with confocal microscopy (Nikon, Japan) or a whole-slide imaging scanner (Hamamatsu, Japan).

Three sagittal brain sections per animal were imaged to evaluate Aβ plaque burden and density. Similar regions encompassing portions of the cortex or hippocampus (CA1 and dentate gyrus) were drawn with the ImageJ software. The Aβ plaque burden was calculated as the ratio of the area occupied by plaque to the total area, while Aβ plaque density was determined as the number of plaques within a given size range for each brain section. The mean plaque density and burden of each animal were obtained by averaging multiple brain sections included in the final count.

Three areas per section were analyzed to determine the number of Aβ plaques associated with microglia. Colocalization was defined as direct contact between microglia and plaques, which was manually identified using the ImageJ software [22]. This identification was performed by persons who were unaware of the experimental grouping.

#### HE staining

Following fixation in 4% paraformaldehyde for 24 h, the brain tissues were dehydrated, embedded, and sectioned. Staining was performed using a kit (Servicebio, China), as previously described [57]. The sections were sealed with neutral gum, and images were captured using a whole-slide imaging scanner (Hamamatsu, Japan).

#### Nissl staining

The brain sections were prepared according to the standard procedure for HE staining. After dewaxing and a distilled water rinse, sections were placed in Nissl staining solution for 2 to 5 min to visualize neuronal cell bodies. Nissl bodies were observed under the microscope as dark blue with a light blue or colorless background, and the staining procedure could be repeated and the time adjusted according to the results. After rinsing with distilled water, the cleared sections were dehydrated and sealed. Images were captured using a whole-slide imaging scanner (Hamamatsu, Japan).

### Quantitative real-time PCR

Total RNA extraction from brain tissue was performed with a kit (Vazyme, China) following the manufacturer’s protocol. First-strand complementary DNA was synthesized from total RNA using a kit (Vazyme, China) according to the manufacturer’s protocol. Quantitative real-time PCR (qPCR) was conducted using an automatic real-time fluorescence qPCR instrument (Roche, Switzerland). Relative changes in gene expression were calculated using the 2^−ΔΔCt^ method. The primer sequences used in this study are listed in Table 1.

**Table 1.** List of primers used for qPCR

Gene	Forward	Reverse
*β-actin*	GAAGTGTGACGTTGACATCCG	GTCAGCAATGCCTGGGTACAT
*RIPK1*	GCCAGTAGCAGATGACCTCA	GCTTGGTGTCTGGAAGTCGA
*TNF-α*	CCACCACGCTCTTCTGTCTACTG	GATGATCTGAGTGTGAGGGTCTGG
*IL-6*	TTCTTGGGACTGATGCTGGTGAC	CTGTTGGGAGTGGTATCCTCTGTG
*IL-1β*	CTCGCAGCAGCACATCAACAAG	CCACGGGAAAGACACAGGTAGC

qPCR, quantitative real-time polymerase chain reaction

### Preparation for oligomer Aβ

Human Aβ_1–42_ peptide and fluorescein isothiocyanate (FITC)-labeled Aβ_1–42_ peptide (GL Biochem Ltd., Shanghai, China) was initially dissolved in 1,1,1,3,3,3-hexafluoro-2-propanol (HFIP) at 1 mM. The peptides were placed in a vacuum pump to evaporate the HFIP at room temperature to create a clear peptide film. Subsequently, dimethyl sulfoxide was added to dissolve the peptide film to form a solution at a concentration of 2 mM, which was diluted to 100 mM in serum-free medium and incubated at 4 °C for 24 h.

### Cell culture and treatment

Culture and maintenance of the BV2 murine microglial cell line were performed in accordance with an established method [58]. BV2 cells were seeded into 96-well plates and divided into 3 groups: blank, oligomer Aβ (oAβ), and oAβ + US groups. Following 24-h incubation, cells were pretreated with Aβ_1–42_ (5 μM) [59,60], followed by 10 min of US applied from beneath the culture plate. The medium containing FITC-Aβ was removed at different time points, and a new medium (without FITC-Aβ) was added after washing the cells with 1× phosphate-buffered saline (PBS). Cells were collected at 24 h for flow cytometry or western blot analysis.

### Flow cytometry

Flow cytometry was utilized to assess the ability of BV2 cells to endocytose FITC-Aβ_1–42_. Cells were collected by centrifugation at 1,000 rpm for 4 min, washed twice with 1× PBS solution, and resuspended in 400 μl of 1× PBS solution. Flow cytometric analysis was immediately performed using the CytoFLEX instrument (Beckman Coulter, USA), and the resulting data were processed with the FlowJo software.

### Golgi staining

Golgi staining was performed using the FD Rapid Golgi Stain Kit (FD NeuroTechnologies, USA). Briefly, mice were deeply anesthetized, and fresh brain tissue was quickly stripped. Liquids A and B were prepared in a 1:1 ratio to immerse brain tissue for 3 weeks at room temperature in the dark. The tissues were transferred to liquid C and immersed for 5 to 7 d at ambient temperature in the absence of light. Coronal brain sections of 100 μm were prepared using a frozen slicer (Leica, Germany) and subjected to Golgi staining following the manufacturer’s instructions. Dendritic spine images were obtained using a 100× objective (Zeiss, Germany), and hippocampal CA1 pyramidal neurons were selected for analysis. Four brain slices were obtained from each mouse, and 5 dendrites were randomly selected from each brain slice to calculate dendritic spine density, and flat values were included in the statistics. The analysis was performed in a blinded manner.

### TUNEL staining

Following the procedures outlined for immunofluorescence staining, brain sections were prepared and processed for terminal deoxynucleotidyl transferase-mediated dUTP nick end labeling (TUNEL) staining using a commercial kit (YEASEN, China) in accordance with the manufacturer’s instructions. To validate the staining protocol, brain sections from middle cerebral artery occlusion model mice were included as a positive control. The stained sections were then imaged using a confocal microscope (Nikon, Japan) and analyzed with the ImageJ software.

### Western blotting

Brain tissue was rapidly removed after mice were perfused via pre-cooled PBS. Cell clumps were obtained by trypsin digestion of cells. Fresh brain tissue or cell clumps were lysed in radioimmunoprecipitation assay buffer (Beyotime, China) containing a protease inhibitor (Servicebio, China). The lysates were then centrifuged to collect the supernatants. The protein concentration was determined according to a previously published protocol with minor modifications [61]. Briefly, during the blocking step, the membranes were incubated with RapidBlock blocking buffer (Bio-Rad, USA) for 5 to 10 min. The primary antibodies used and their corresponding dilution ratios are listed in Table 2. Protein signals were detected by incubating the membranes with an enhanced chemiluminescence substrate (Bio-Rad, USA) followed by visualization in a gel documentation system (Bio-Rad, USA). Protein bands were analyzed with the ImageJ software. In some cases, the bands were stripped in a quick stripping solution (Epizyme, China) for 10 min at room temperature with gentle shaking before reprobing with an additional primary antibody.

**Table 2.** List of primary antibodies used for western blotting

Primary antibody	Species	Conc.	Company
Phosphorylated *RIPK1*	Rabbit	1:1,000	Cell Signaling (31122)
*RIPK1*	Rabbit	1:1,000	Cell Signaling (3493)
Phosphorylated *RIPK3*	Rabbit	1:1,000	HUABIO (HA721428)
*RIPK3*	Rabbit	1:1,000	HUABIO (ER1901-27)
Phosphorylated *MLKL*	Rabbit	1:1,000	Cell Signaling (37333)
*MLKL*	Rabbit	1:1,000	ABclonal (A21894)
Phosphorylated NF-κB	Rabbit	1:1,000	Cell Signaling (30337)
NF-κB	Rabbit	1:1,000	Cell Signaling (8242)
*TNF-α*	Rabbit	1:1,000	abcom (6671)
*IL-1β*	Rabbit	1:1,000	Cell Signaling (31202)
*IL-6*	Rabbit	1:1,000	Cell Signaling (12912)
*PSD95*	Rabbit	1:10,000	abcom (18258)
*SYN*	Rabbit	1:10,000	abcom (EP1098Y)
*β-actin*	Rabbit	1:5,000	abcom (8227)
*GAPDH*	Rabbit	1:5,000	Cell Signaling (5174)

### RNA sequencing

Total RNA was extracted using the Total RNA Extractor (TRIzol) kit (Sangon, China). The quality of RNA samples was assessed using a NanoPhotometer spectrophotometer. High-quality RNA samples were subsequently submitted to Sangon Biotech (Shanghai) for complementary DNA library construction, RNA-seq, and data analysis. DESeq2 (version 1.12.4) was employed to identify DEGs between the compared groups. DEGs were defined by a *q*-value threshold of ≤0.001 and an absolute fold change ≥2. For functional enrichment analysis, GO terms and KEGG pathway with a false discovery rate (*q* value) <0.05 were considered as significantly changed.

### Statistical analyses

Statistical analyses were performed using GraphPad Prism (version 9.5). The normality of data distribution was verified with the Shapiro–Wilk test. For comparisons between 2 groups meeting normality assumptions, the Student *t* test was applied to assess statistical significance. Statistical comparisons across multiple groups were performed using one-way analysis of variance for normally distributed data, followed by the Kruskal–Wallis test for nonparametric datasets. All results are expressed as mean ± standard error of the mean, and a *P* value <0.05 was considered statistically significant.

## Ethical Approval

All animal care and experiment protocols were approved by the Animal Experiment Ethics Committee of the Shenzhen Institutes of Advanced Technology, Chinese Academy of Sciences (SIAT-IACUC-230103-YGS-NLL-A2241).

## Data Availability

The data that support the findings of this study are available from the corresponding authors upon reasonable request.
