# Supplementary material for: Transcranial Ultrasound Stimulation Pulsed at 40 Hz Improves Cognition and Neuroinflammation in Female Mice with Alzheimer’s Disease
Source: Research (Wash D C). 2026 Apr 20;9:1244. doi: 10.34133/research.1244 (PMC13093894; doi:10.34133/research.1244)
Supplement: Supplementary 1 — Figs. S1 to S8 [file research.1244.f1.zip › Supplementary Materials.docx]

**Supplementary Materials**


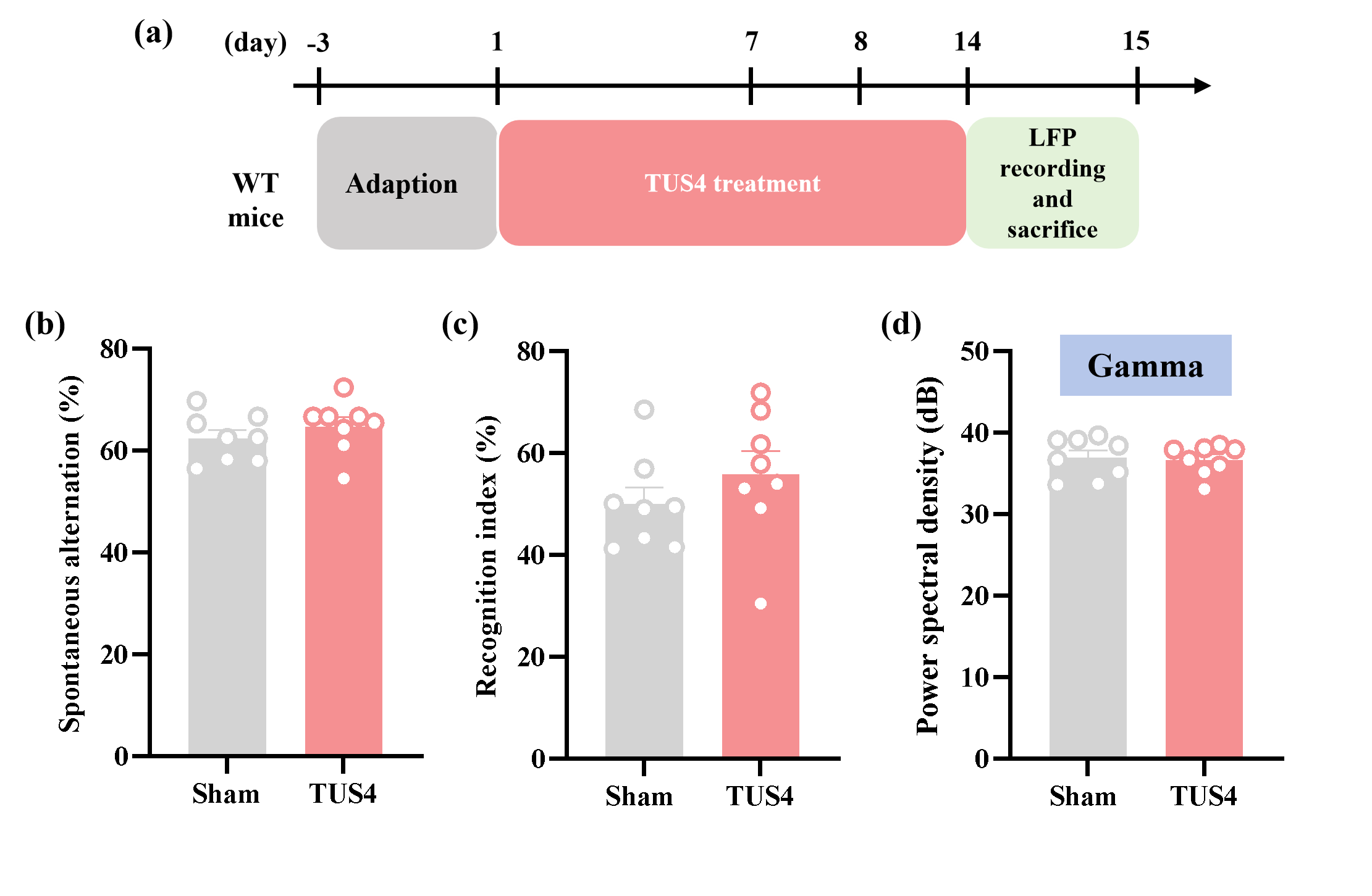


**Supplementary Figure 1.** Effects of TUS4 on cognitive function and hippocampal LFP in WT mice. (a) Experimental time flow diagram. (b) Quantitative analysis of spontaneous alteration in the Y‑maze test across groups. (c) Quantitative analysis of recognition index in test period of NOR across groups. (d) Quantitative analysis of average power change in gamma bands across groups. WT, wild-type.


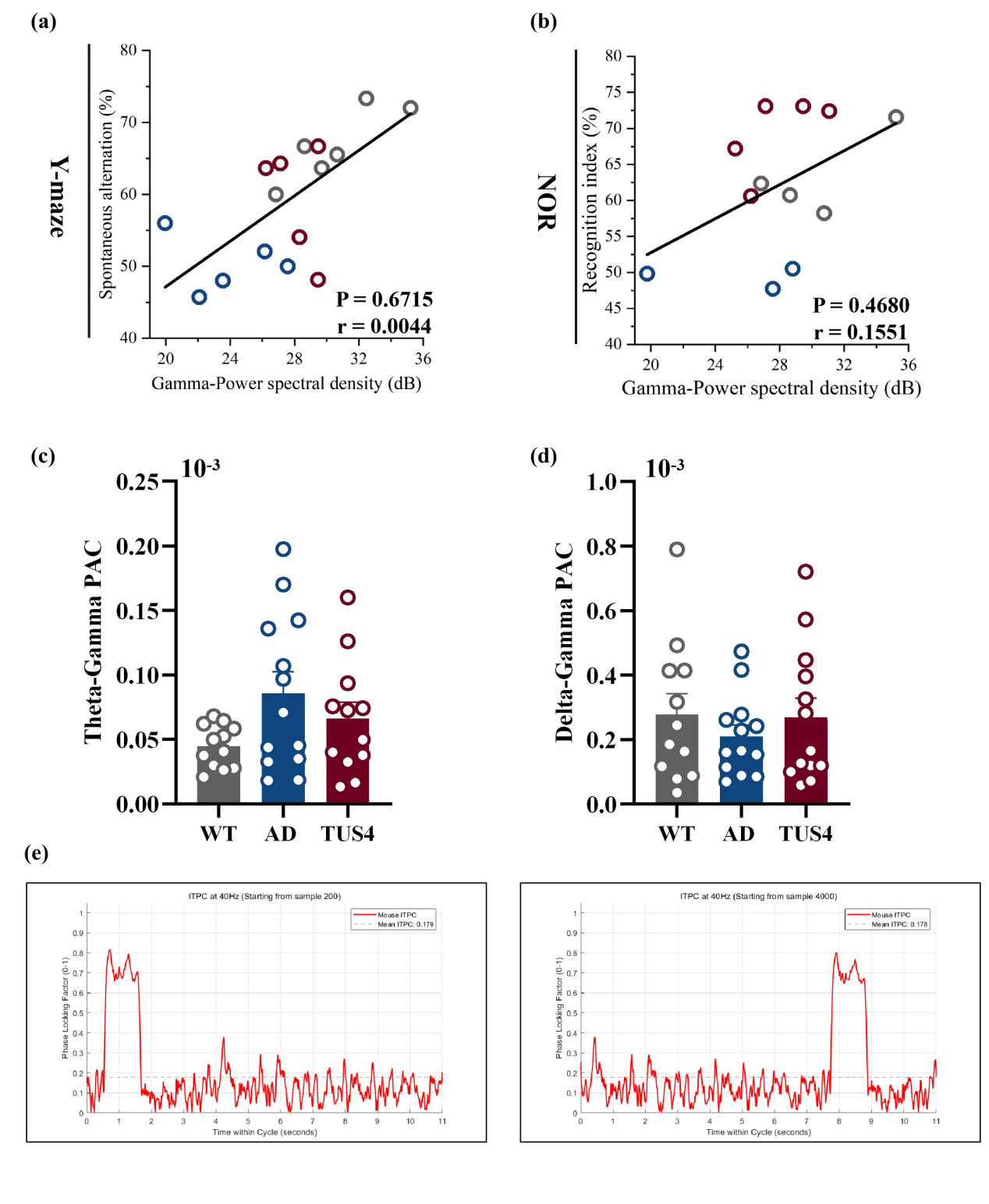


**Supplementary Figure 2.** Analysis of neural oscillations and phase entrainment. (a-b) Correlation between hippocampal gamma power and behavioral performance (Y-maze and NOR). (c-d) Statistical analysis of cross-frequency phase-amplitude coupling for delta-gamma and theta-gamma pairs across experimental groups. (e) ITPC by aligning LFP trials to the phase of the 40 Hz ultrasonic cycles, analyzed from two different post-recording starting points (200 and 4000 sampling points). NOR, novel object recognition; ITPC, calculation of inter-trial phase coherence; LFP, local field potentials.


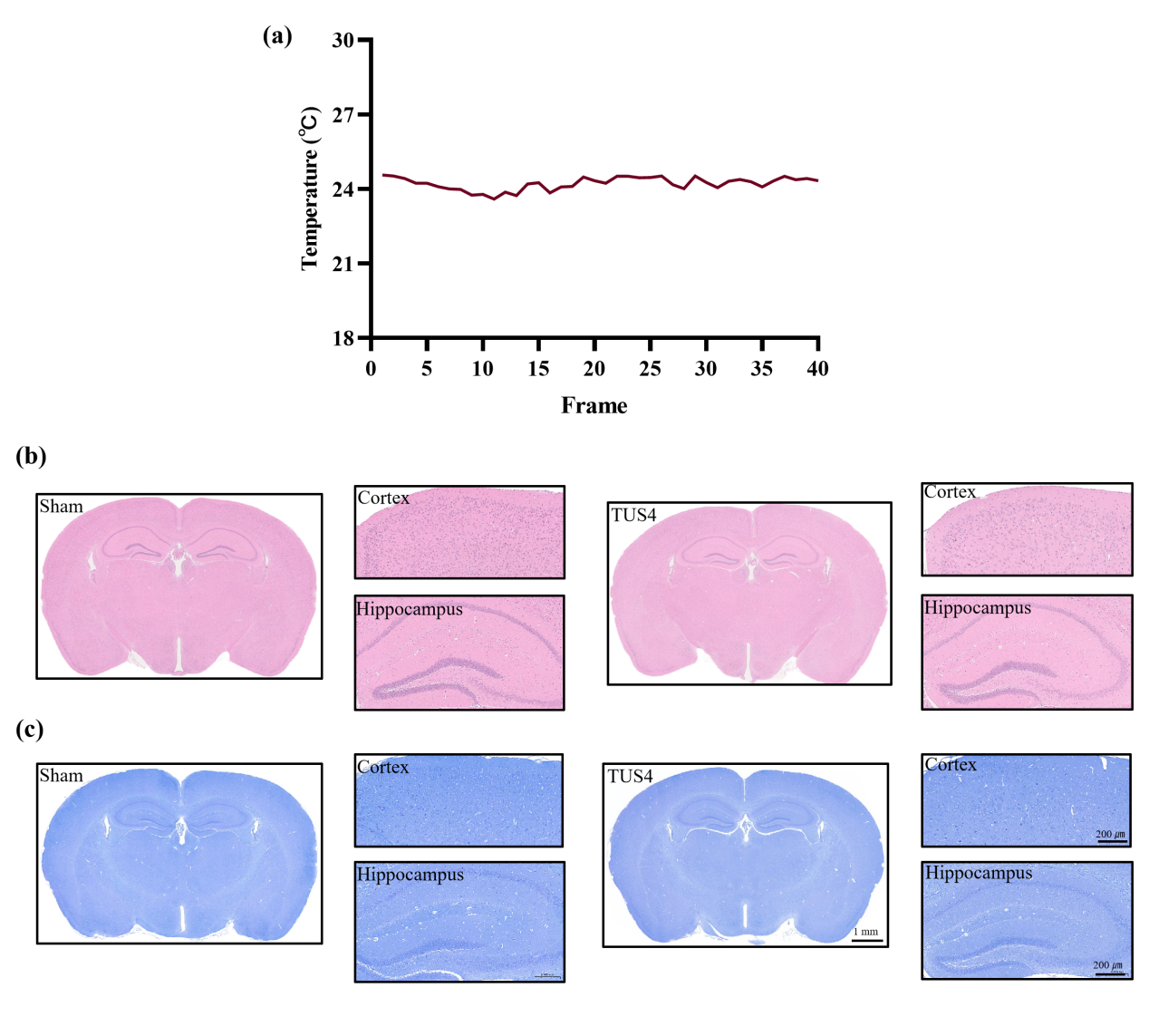


**Supplementary Figure 3.** Thermal effects and safety assessment of TUS. (a) Statistical maps of cranial temperature changes during 20 min of TUS4 treatment (one frame of image acquired at 30 s). (b) Representative graphs of HE staining of mice in Sham and TUS4 groups. (c) Representative graphs of Nisll staining of mice in Sham and TUS4 groups.

**
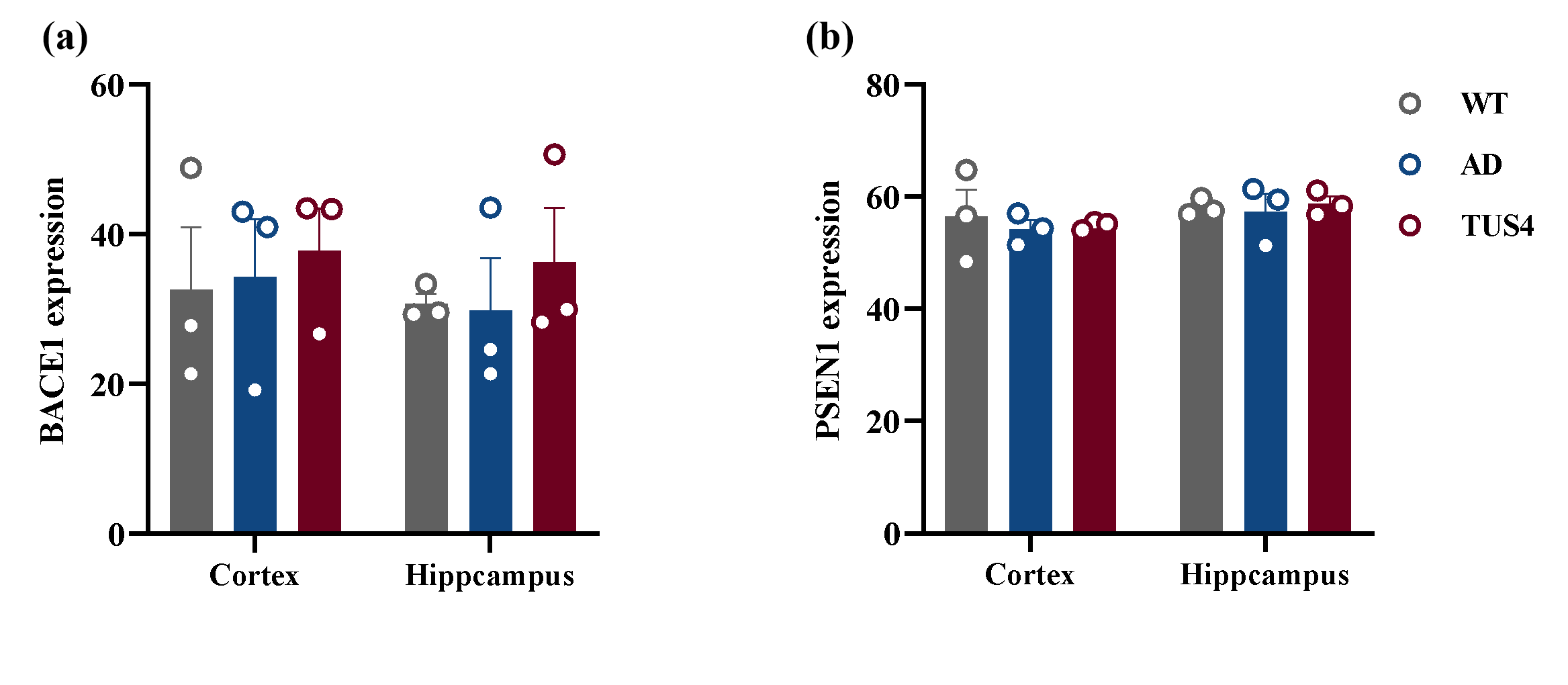
**

**Supplementary Figure 4.** (a) Comparison of BACE1 gene expression between WT, AD and TUS4 group. (b) Comparison of PSEN1 gene expression between WT, AD and TUS4 group.

**
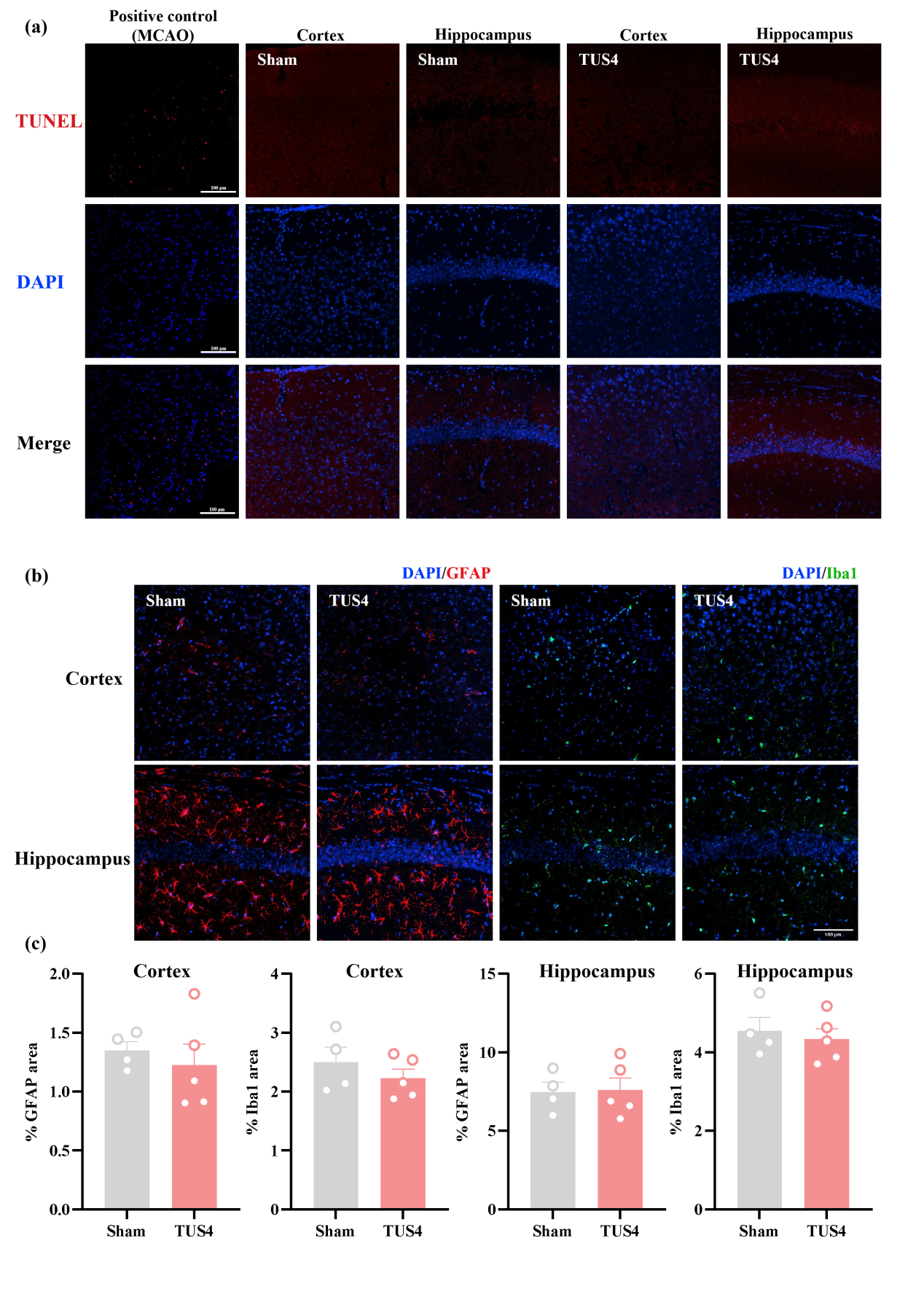
**

**Supplementary Figure 5.** Histological safety assessment following TUS. (a) Representative images of TUNEL staining for detecting apoptotic cells in positive control (MCAO), Sham and TUS4 mice. (b) Representative immunofluorescence images for microglia (Iba1) and astrocytes (GFAP). (c) Quantification of the percentage of GFAP and Iba1 immunopositive areas relative to the total image area. MCAO, middle cerebral artery occlusion.


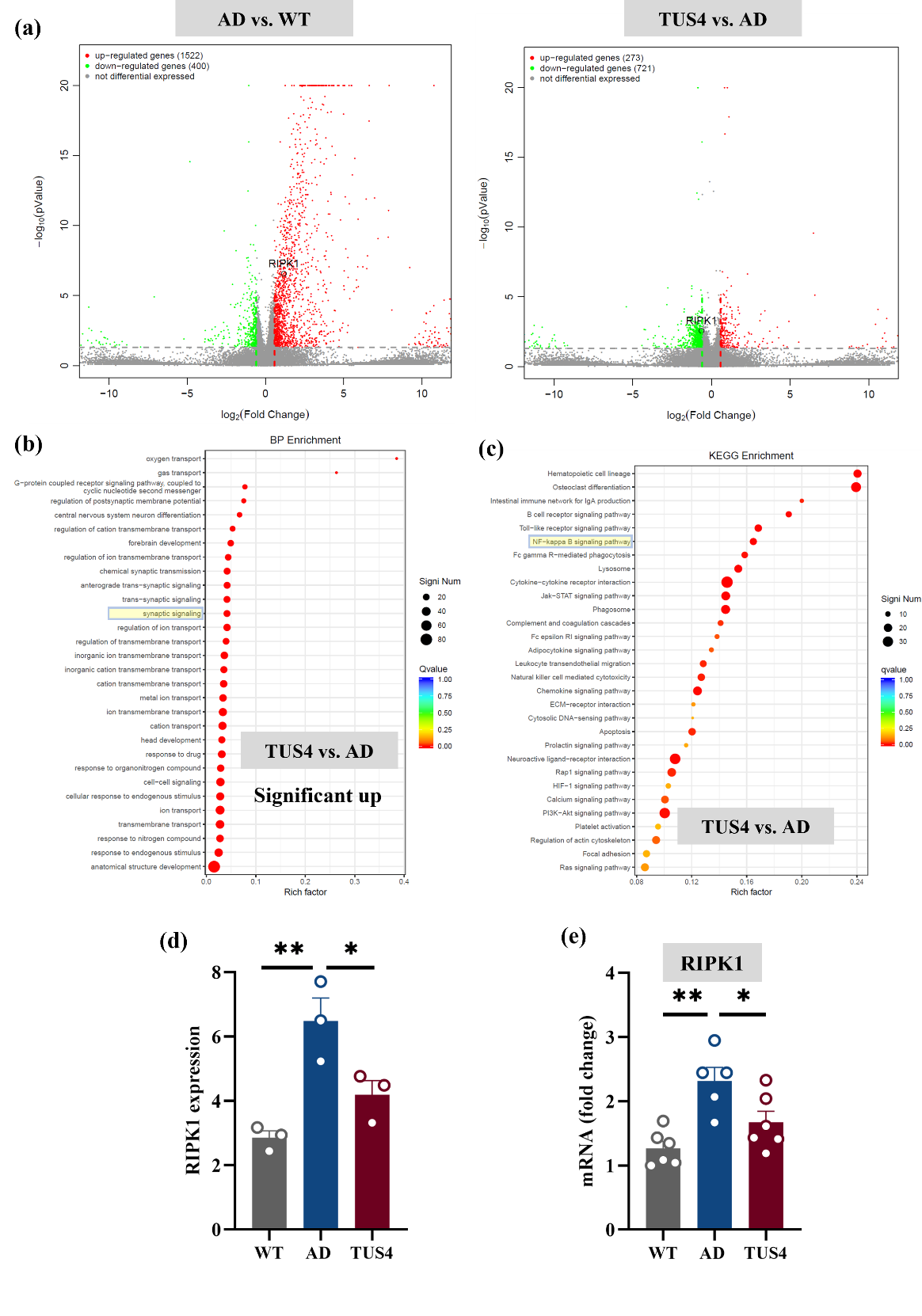
**Supplementary Figure 6.** The results of RNA-seq. The effect of TUS treatment on (a) Corresponding volcano plot of AD group versus WT group and TUS4 group versus AD group. (b) The GO enrichment analysis of the differentially expressed genes in TUS4 vs. AD group, the most significant 30 terms of Biological Process were selected to draw a scatter diagram for display. (c) The KEGG pathway analysis of differentially expressed genes in TUS4 vs. AD group, the most significant 30 terms were selected to draw a scatter diagram for display. (d) Comparison of RIPK1 gene expression between WT, AD and TUS4 group. (e) Quantitative analysis of RIPK1 expression of different group in RT-qPCR. **P* < 0.05, ***P* < 0. 01.


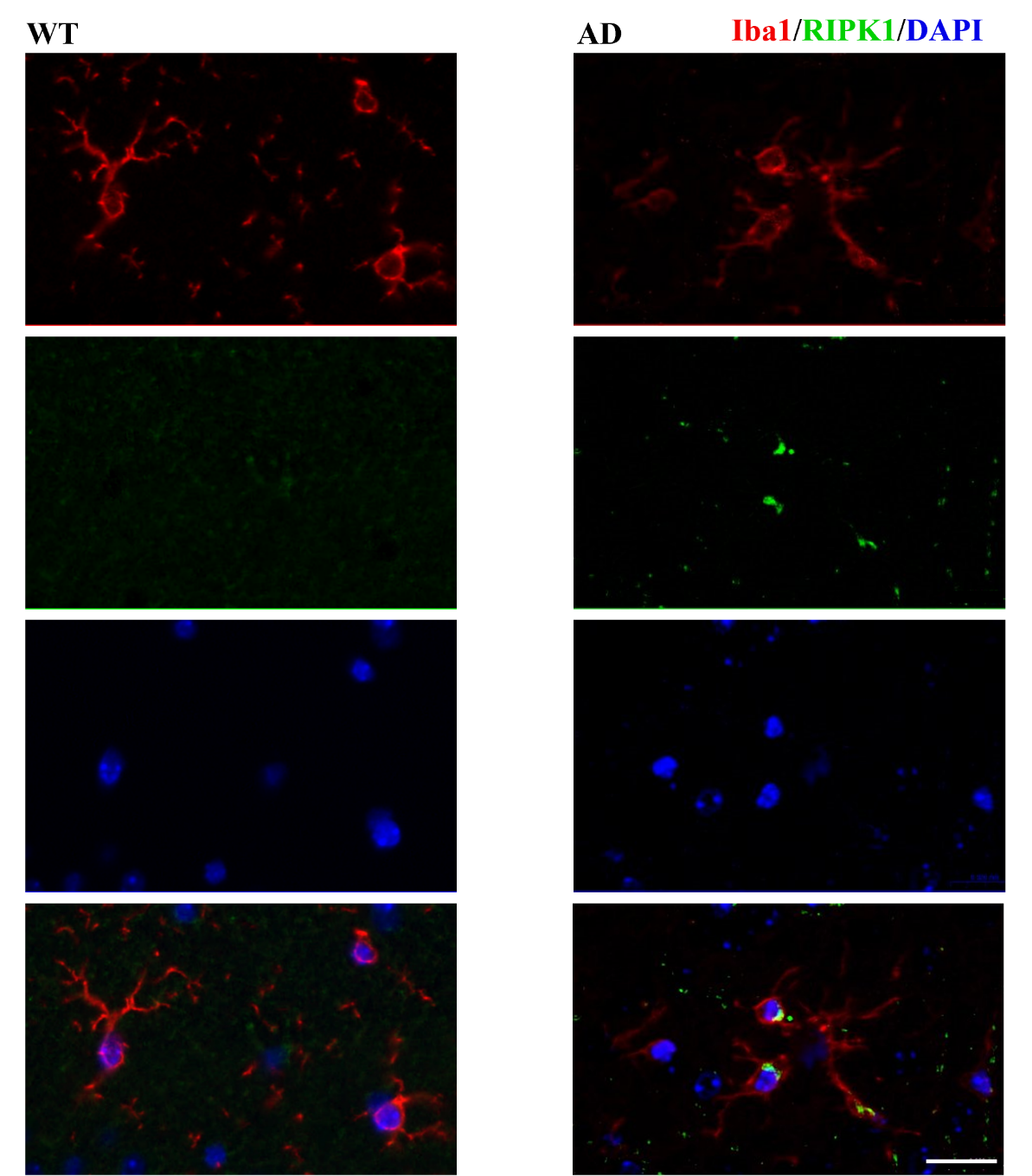


**Supplementary Figure 7**. Immunofluorescence staining showed that highly expressed RIPK1 in the hippocampus of AD mice localizes to microglia. Scale bars: 20 μm.


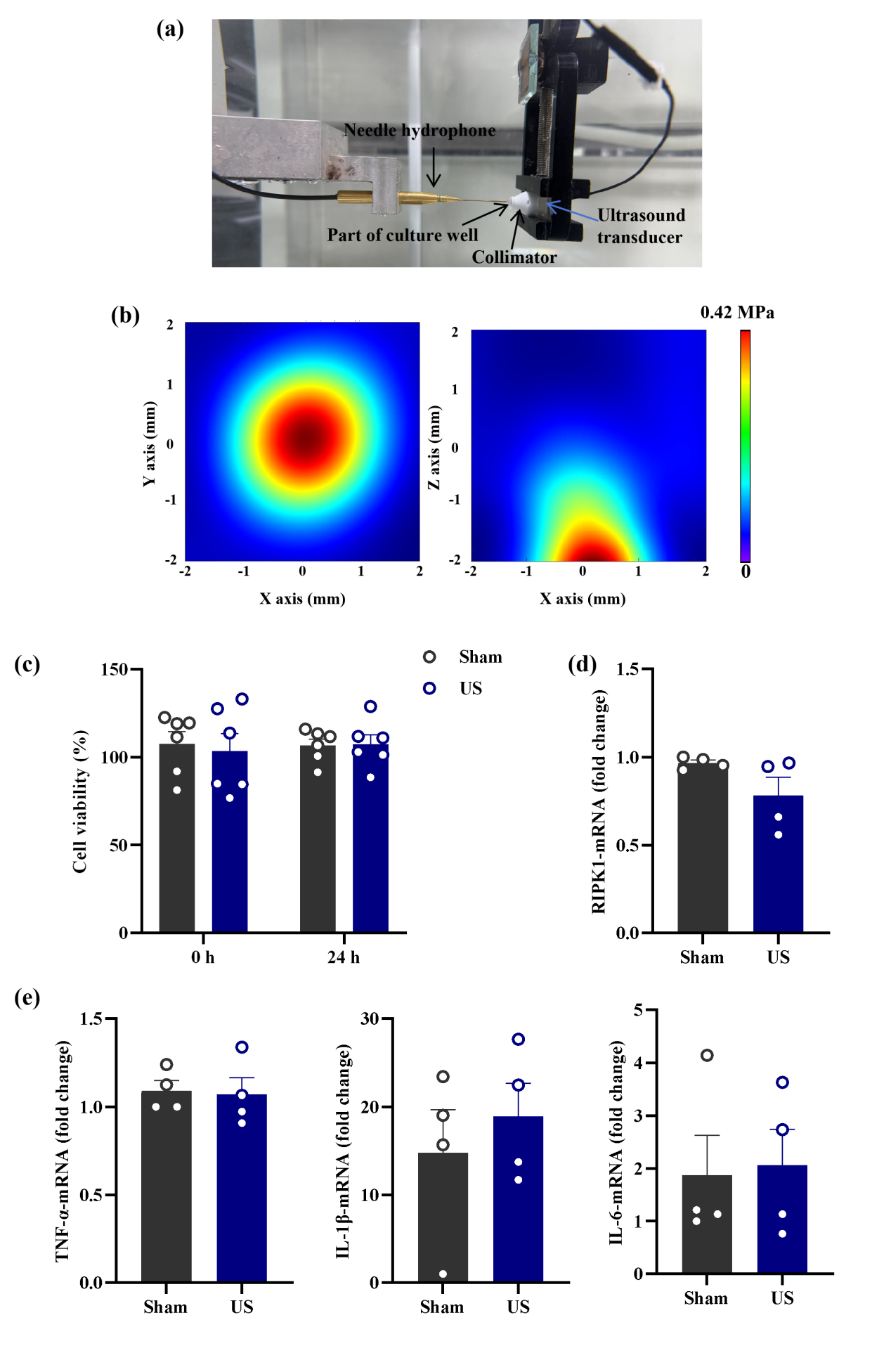


**Supplementary figure 8**. US has no significant effect on intrinsic signaling in BV2 microglia in the absence of Aβ. (a) Photograph of the acoustic field generated by ultrasound waves passing through a cell culture plate. (b) Measured distribution of the acoustic pressure field within the culture well. (c) Cell viability assessed at 0 and 24 hours after US. (d) Relative mRNA levels of Ripk1 in BV2 cells across experimental groups. (e) Relative mRNA levels of the pro-inflammatory cytokines TNF-α, IL-1β, and Il-6 in BV2 cells across experimental groups. US,ultrasound stimulation.
