# Supplementary figures and images for: Transcranial Ultrasound Stimulation Pulsed at 40 Hz Improves Cognition and Neuroinflammation in Female Mice with Alzheimer’s Disease
Source: Research (Wash D C). 2026 Apr 20;9:1244. doi: 10.34133/research.1244 (PMC13093894; doi:10.34133/research.1244)

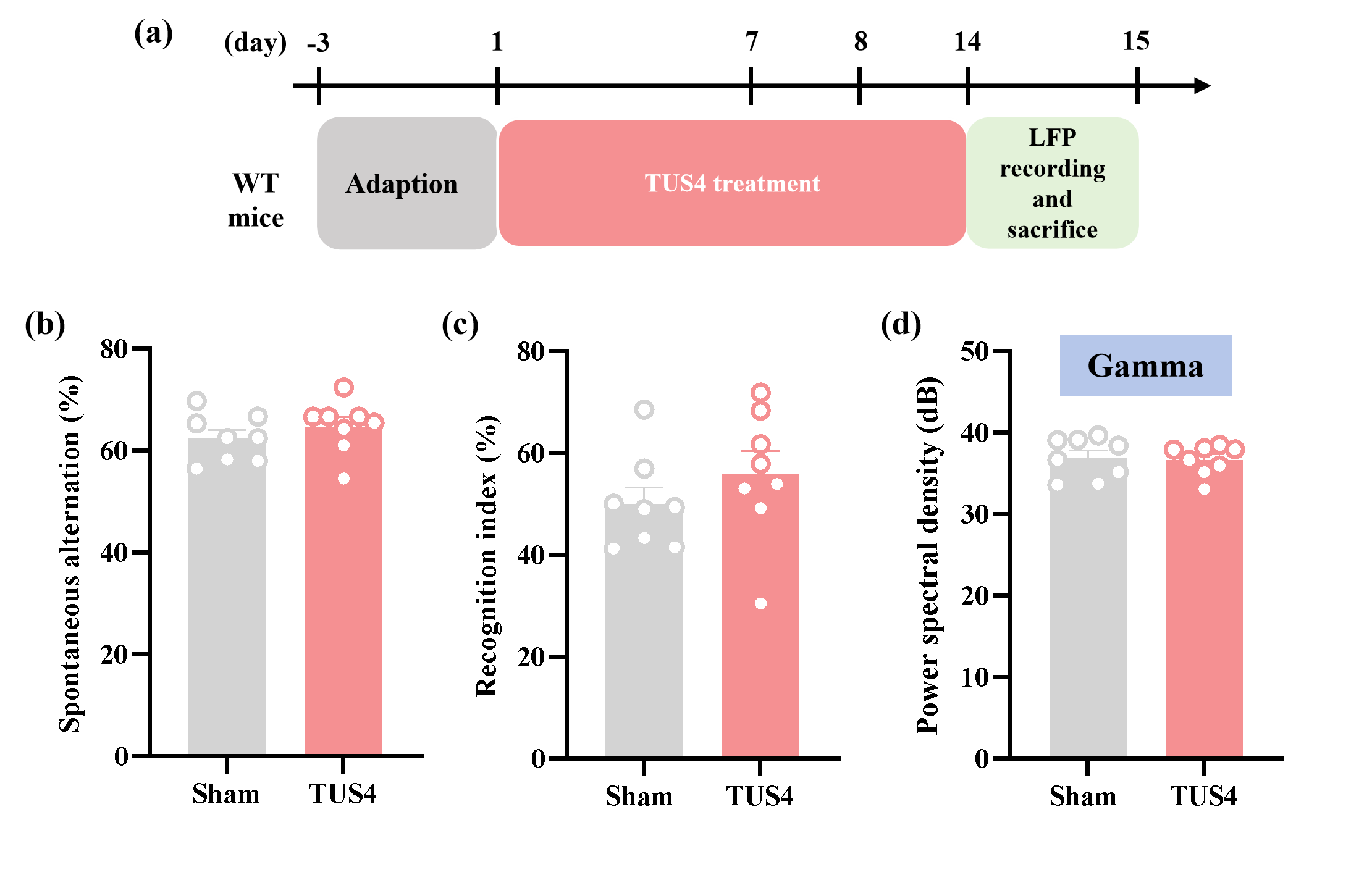

Supplement: Supplementary 1 — Figs. S1 to S8 [file research.1244.f1.zip › Supplementary Figure1.tif]

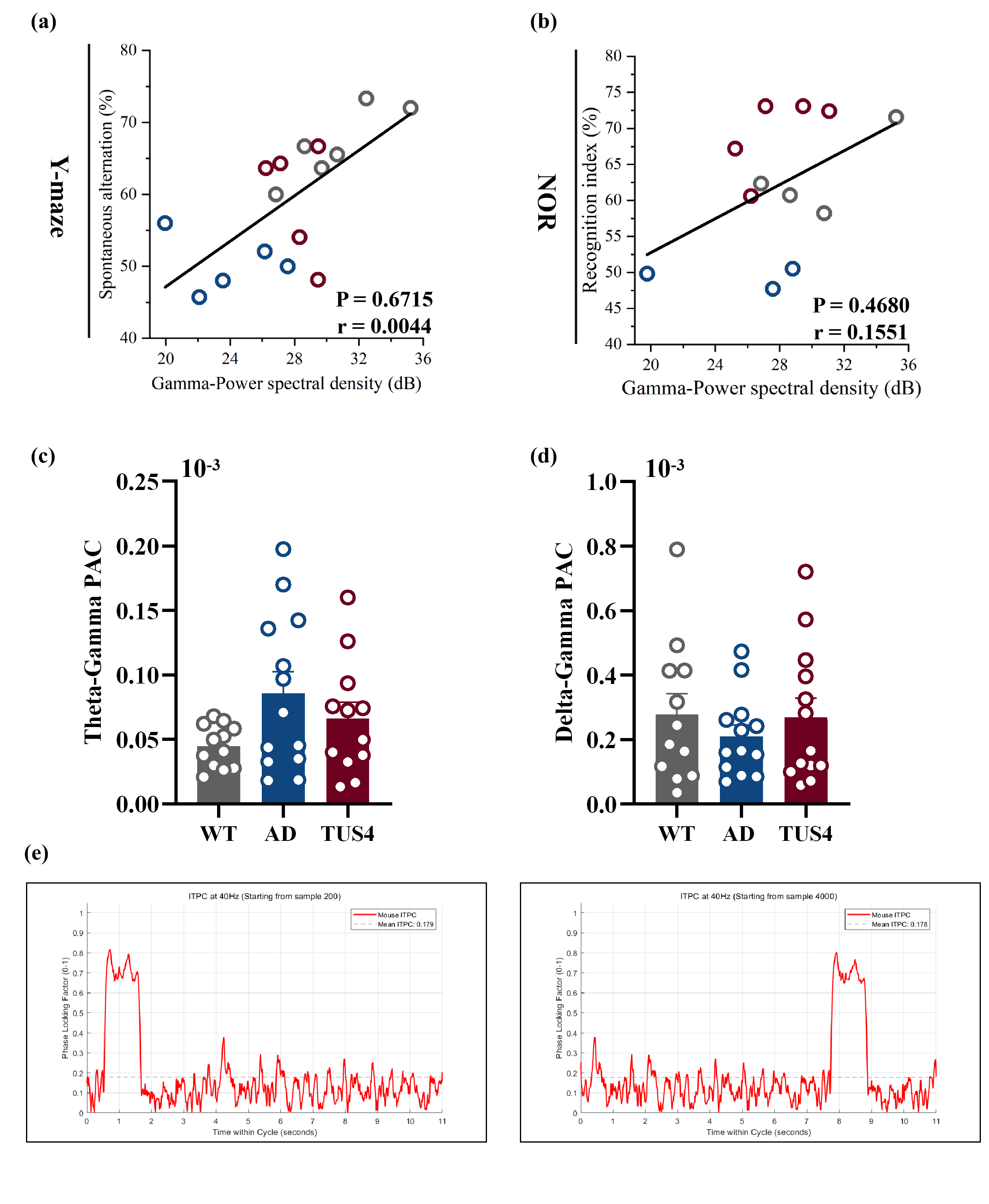

Supplement: Supplementary 1 — Figs. S1 to S8 [file research.1244.f1.zip › Supplementary Figure2.tif]

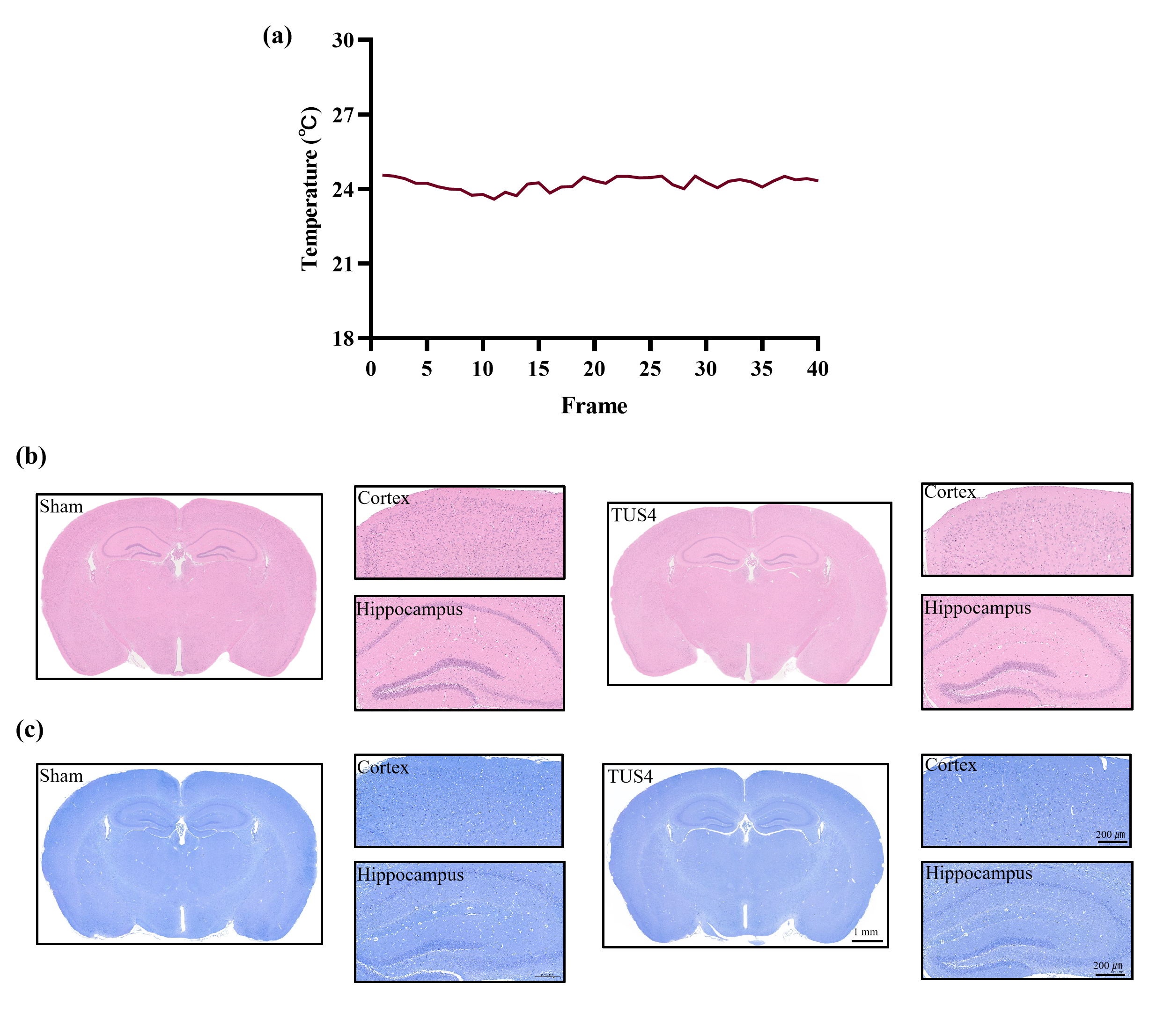

Supplement: Supplementary 1 — Figs. S1 to S8 [file research.1244.f1.zip › Supplementary Figure3.tif]

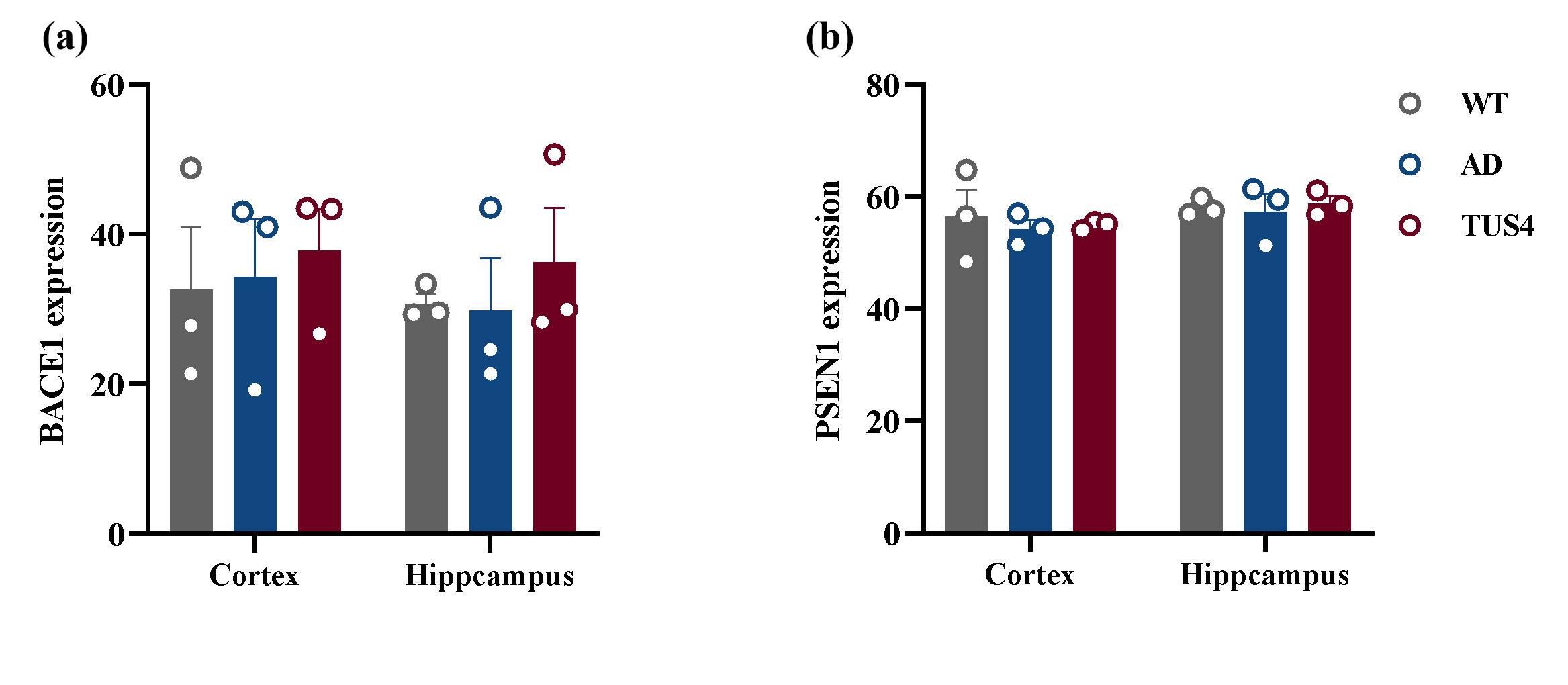

Supplement: Supplementary 1 — Figs. S1 to S8 [file research.1244.f1.zip › Supplementary Figure4.tif]

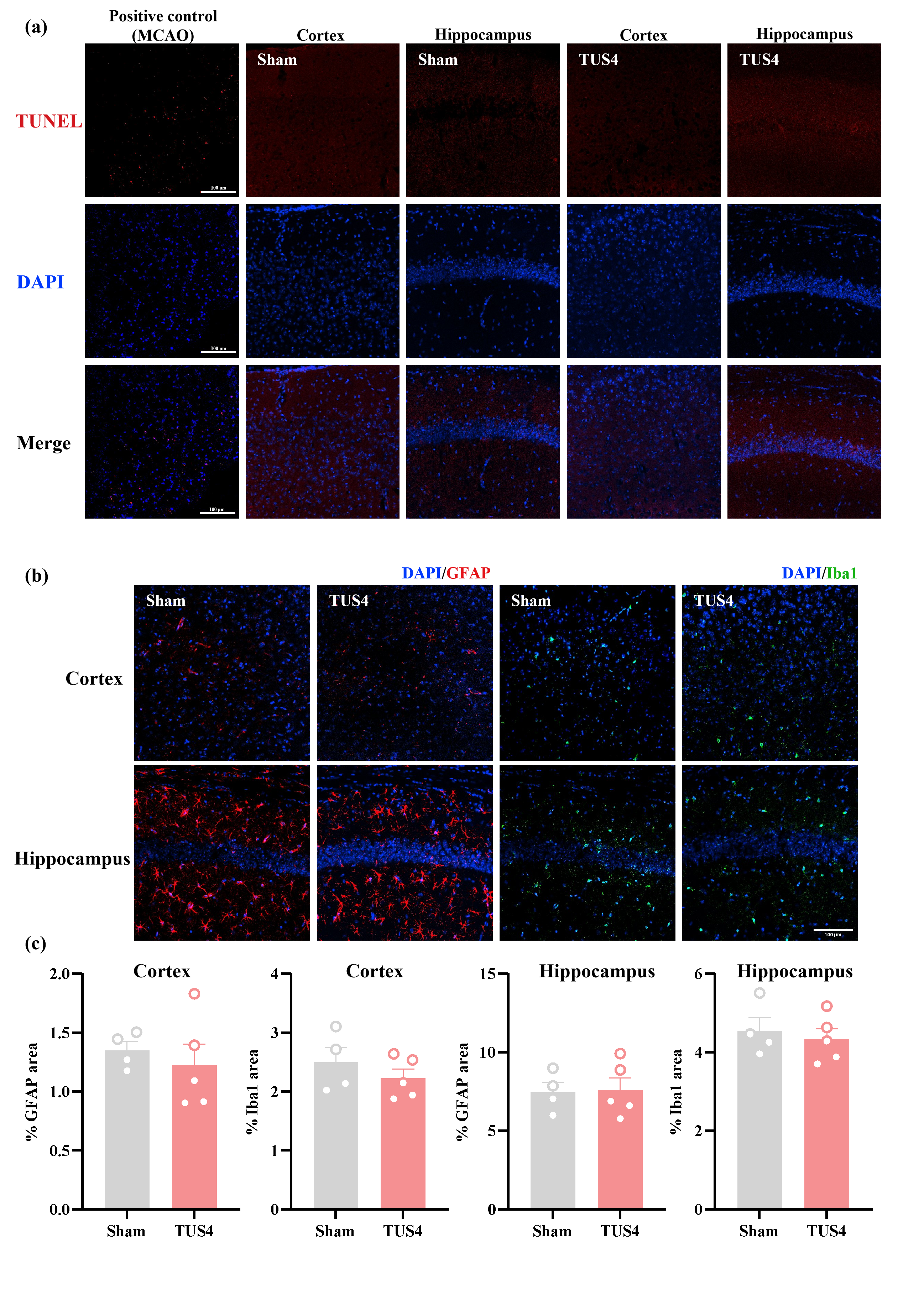

Supplement: Supplementary 1 — Figs. S1 to S8 [file research.1244.f1.zip › Supplementary Figure5.tif]

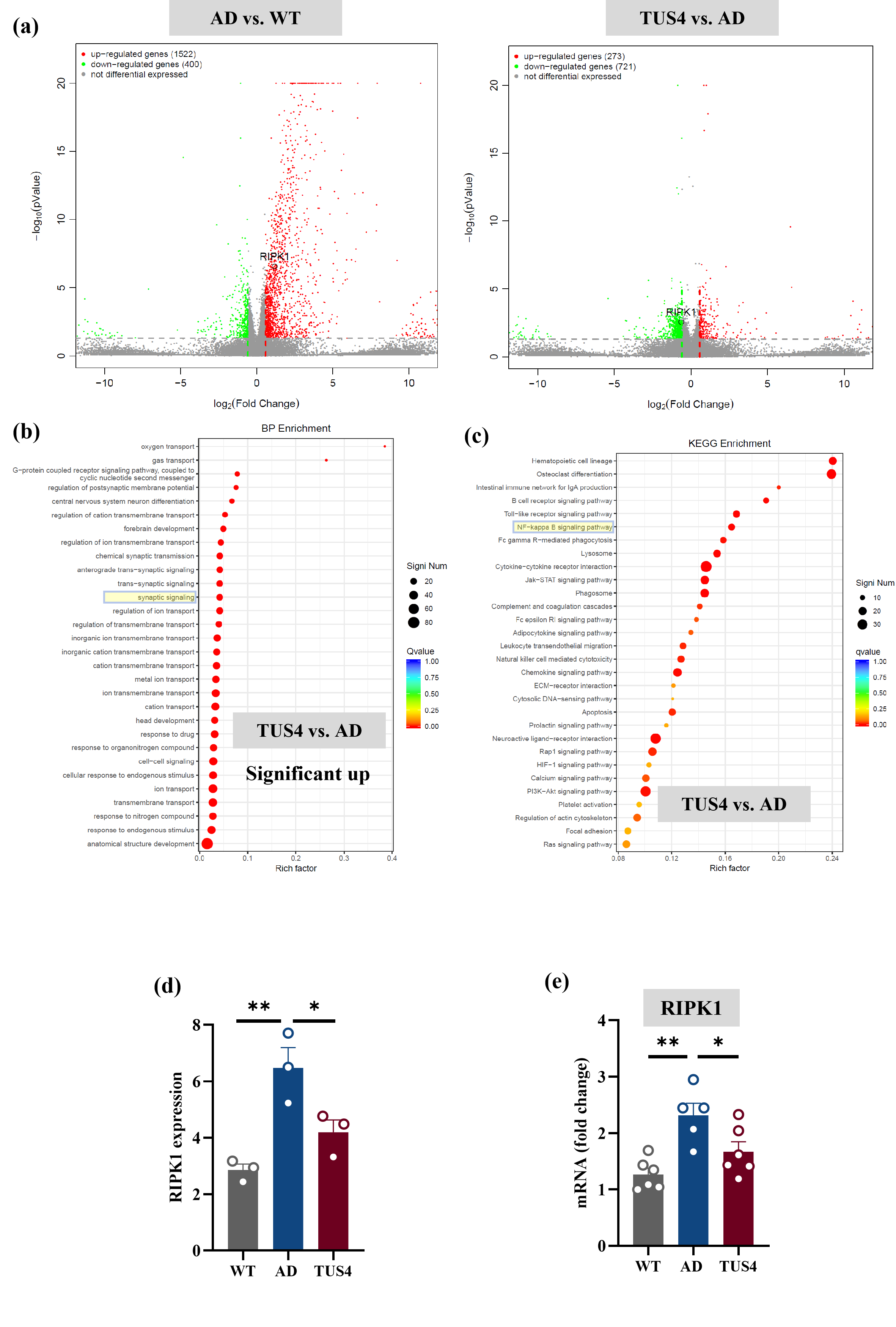

Supplement: Supplementary 1 — Figs. S1 to S8 [file research.1244.f1.zip › Supplementary Figure6.tif]

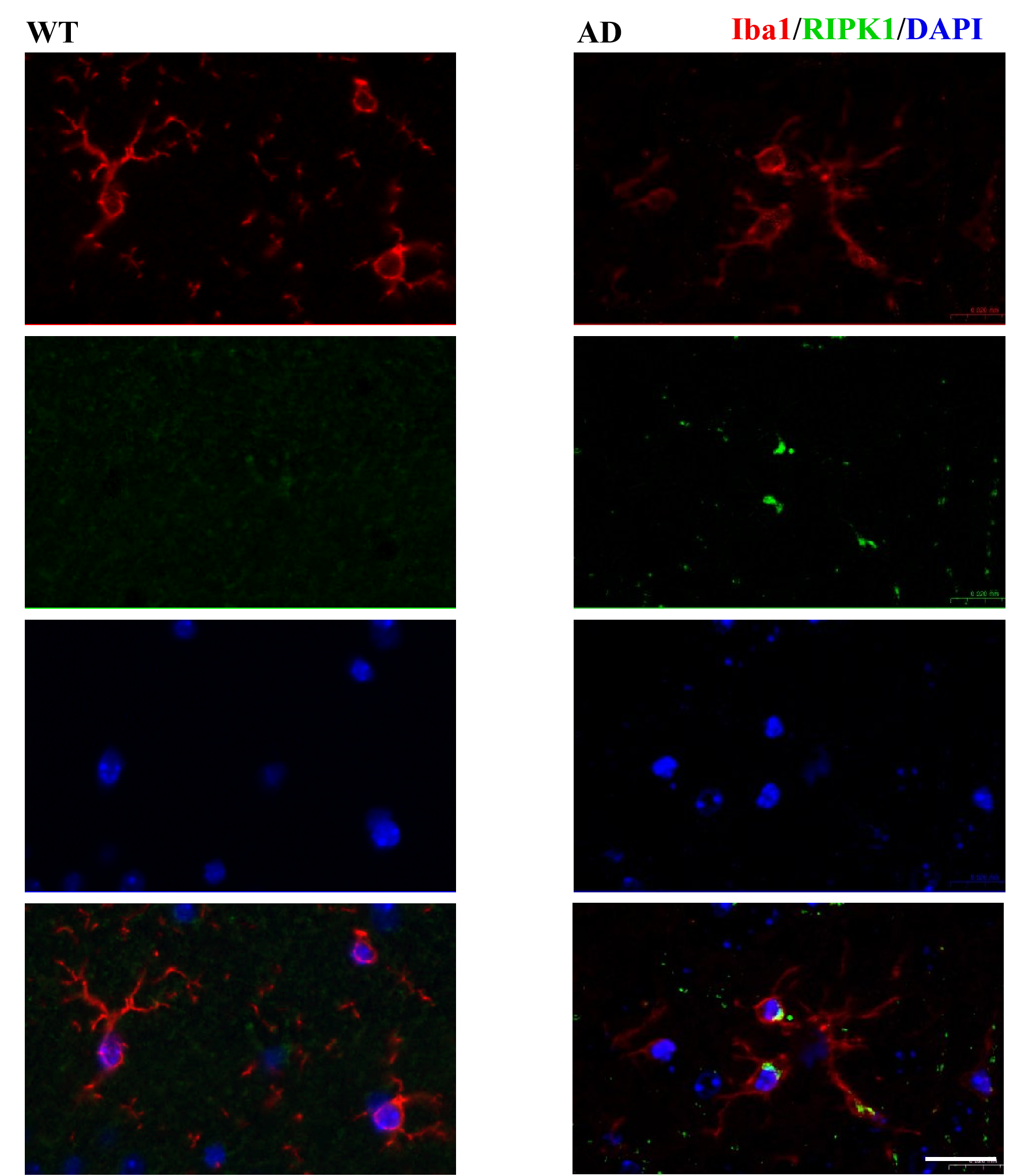

Supplement: Supplementary 1 — Figs. S1 to S8 [file research.1244.f1.zip › Supplementary Figure7.tif]

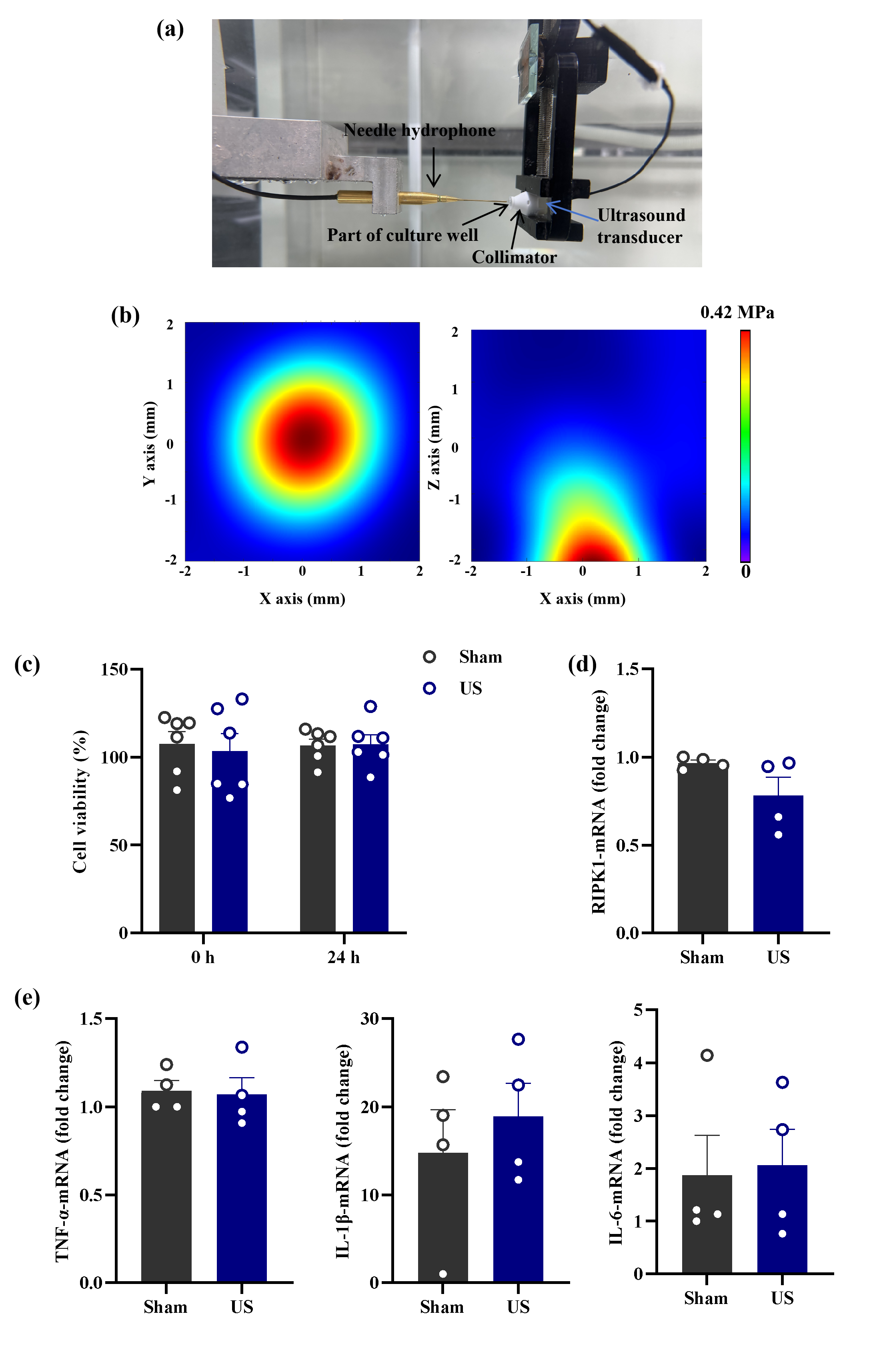

Supplement: Supplementary 1 — Figs. S1 to S8 [file research.1244.f1.zip › Supplementary Figure8.tif]
